# Accurately quantifying the shape of birds’ eggs

**DOI:** 10.1002/ece3.4412

**Published:** 2018-09-05

**Authors:** John D. Biggins, Jamie E. Thompson, Tim R. Birkhead

**Affiliations:** ^1^ School of Mathematics and Statistics The University of Sheffield Sheffield UK; ^2^ Department of Animal and Plant Sciences The University of Sheffield Sheffield UK

**Keywords:** asymmetry, elongation, guillemot, pointedness, pyriform, shape indices, shape parameters

## Abstract

Describing the range of avian egg shapes quantitatively has long been recognized as difficult. A variety of approaches has been adopted, some of which aim to capture the shape accurately and some to provide intelligible indices of shape. The objectives here are to show that a (four‐parameter) method proposed by Preston (1953, *The Auk*, 70, 160) is the best option for quantifying egg shape, to provide and document an R program for applying this method to suitable photographs of eggs, to illustrate that intelligible shape indices can be derived from the summary this method provides, to review shape indices that have been proposed, and to report on the errors introduced using photographs of eggs at rest rather than horizontal.

## 1 INTRODUCTION

Birds’ eggs occur in a remarkable range of sizes and shapes, from almost spherical to extremely elongate and symmetrical to pointed at one end (pyriform). The causes and evolutionary consequences of interspecific differences in avian egg shape have puzzled biologists for over a century, yet the way different egg shapes are produced within the oviduct and the adaptive significance of egg shape remain largely unresolved. One reason for this has been the difficulty of quantifying egg shape, as no single index captures effectively the full range of avian egg shapes or, indeed, other taxa such as reptiles (Birkhead, Thompson, Jackson, & Biggins, 2017; Deeming & Ruta, 2014; Stoddard et al., 2017).

It is convenient to speak of the two pointed ends of the egg as poles, giving a natural sense to “latitude” (distance between the poles along the line joining them), “equator” (the points on the surface at equal distance from the two poles), and “meridian” (the profile of the surface from pole to pole). In an early study of avian egg shape, Mallock (1925) examined the implications of the observation that eggs have circular latitudinal cross‐sections. It is this observation that justifies capturing egg shape through a suitable formula for the meridian and means that from this, together with a length measurement, any characteristic, such as volume or surface area, of the egg shape can be obtained. Thus, although the focus here is on avian egg shapes, the methods would apply also to eggs of other taxa that have circular cross‐sections. Several authors (Mallock, 1925; Okabe, 1952; Stoddard et al., 2017; Thompson, 1942) have considered the mechanisms by which different egg shapes might be achieved. We have no additional insights into that topic, which is distinct from seeking a simple accurate summary for the shape.

Romanoff and Romanoff (1949, p88) state “the numerous variations in the contour of individual eggs obviously cannot be expressed in mathematical terms” and, commenting on Thompson's (1942) magnificent treatise “On Growth and Form”, Preston (1953, p160) said that Thompson “seemed to throw up his hands in the belief that egg shape is indescribable, particularly if it happens to be a guillemot's (=murre's)” [i.e. *Uria aalge*]. Preston (1953) went on to propose an approach that captures the whole range of the shapes of eggs through four parameters. This insight underpins the subsequent studies by Preston (1968) and Todd and Smart (1984).

Although Preston (1953) solved the problem of capturing egg shape, the parameters he employed do not have a simple intuitive relation to the most striking aspects of shape. Thus, other, more direct, measurements have been proposed (see Section 2.6 below for further discussion). In particular, informed by his earlier insights, Preston (1968) identified three indices (which he calls asymmetry, bicone and elongation) that he considered captured the variation in avian egg shape, including the pyriform (pointed) eggs of birds such as the guillemot. However, this set of indices has not been widely adopted, for three reasons: (a) two of his indices (asymmetry and bicone) depend on a measure of the curvature at the ends of an egg that he obtained using a specially constructed device (a spherometer); (b) the two indices derived using the spherometer are, as Preston explains, motivated by, but are not the same as, other indices that are more directly related to the fitted shape but less practical to measure; (c) his mathematical formulations may have deterred some researchers from exploring his ideas (see also Mänd, Nigul, & Sein, 1986, p613).

Instead, researchers have often used just two simpler indices: (a) asymmetry – the extent to which the latitude with widest breadth deviates from the equator; (b) elongation – length relative to breadth. Neither of these is precisely specified by these descriptions and a number of variants exist (see Section 2.6 below). Thus, the potential for confusion is considerable: the same shape index is sometimes referred to by different names by different authors, and in some cases, different shape indices are referred to by the same name. In general, indices are not methods for capturing egg shape accurately in all cases, but, rather, are ways of obtaining certain summary measures that are intuitively related to key aspects of shape.

An important aspect of these different measures of “asymmetry” and “elongation” is that they all fail to deal satisfactorily with eggs of certain shapes, in particular pyriform eggs produced by some alcids and waders (shorebirds). For example, the recent wide‐ranging comparative study by Stoddard et al. (2017) uses two indices, based on Baker's (2002) formulations. However, this method did not quantify the shape of pyriform eggs sufficiently accurately and so they were excluded from their analysis (see figure S2 in Stoddard et al., 2017).

Digital photography and the automated handling of the digital images mean that the field constraints that influenced Preston's (1968) choice of summary indices no longer apply. Now, instead, Preston's (1953) original ideas for summarizing egg shape can be applied automatically — a possibility he anticipated (Preston, 1969; p262–3). The software we have developed (see the Supporting Information: Supplementary Material,^1^ Section SupM5), which works best with egg silhouettes, does this.

The studies by Barta and Székely (1997), Mónus and Barta (2005) and Bán et al. (2011) are already in this vein, working from photographs, except they did not process images automatically, and, presumably as a consequence, used a limited number of points on the meridian in the curve fitting. Mityay, Matsyura, and Jankowski (2015) do seem to have processed a large number of photographs and fitted Preston parameters, although these are, rather misleadingly, attributed to Frantsevich (2015) rather than Preston (1953). Moreover Mityay et al. (2015) give no detail of their fitting methods. Attard, Medina, Langmore, and Sherratt (2017) processed egg images automatically by drawing on sophisticated Fourier techniques designed to capture even very complicated closed contours, to produce a large set of coefficients and then reduced the coefficient set using principal components. However, egg profiles are really very simple closed contours, as the success of Preston's approach shows, which can be summarized much more directly.

Alternative methods of summarizing egg shape have been proposed by Carter (1968), Carter and Morley Jones (1970), Baker (2002) and Troscianko (2014). When compared with Preston's (1953) proposal, each of these is less effective in capturing egg shape for some eggs (see Sections 3.1 and 3.2 below). Several other possible mathematical forms have been identified, as the web pages maintained by Köller (2017) illustrate. In particular, Thompson (1942, p936) mentions the Cartesian Oval as a proposal going back to the middle of the nineteenth century, although he points out that this proposal “fails in such a case as the guillemot.”

The aims here are to: (a) enable, via the accompanying software, the automated use of Preston's (1953) original proposal for capturing egg shape, and to extend it somewhat; (b) illustrate that that proposal has sufficient flexibility to capture very accurately the shape of all eggs including pyriform eggs and that the methods of Carter (1968), Carter and Morley Jones (1970), Baker (2002) and Troscianko (2014) are less effective; (c) show that egg positioning for the photographs matters; (d) illustrate that once Preston's parameter's and the length of the egg are available, any characteristic of the egg shape and size can be obtained – in particular, three interpretable indices of shape: Elongation, Pointedness, and Polar Asymmetry (described in Section 2.5); and (e) present a review of the various measures of egg shape that have been used previously and their relationships and demonstrate the appropriateness of the indices Elongation, Pointedness and Polar Asymmetry for describing the shape of pyriform eggs.

## 2 METHODS

### 2.1 Formulae for egg shape

Imagine an egg with its longest axis horizontal, on the *x*‐axis, and with the length scale arranged so that the two poles are at −1 and 1, which means the egg's length is scaled to be two. The height of the egg outline above the horizontal axis at *x* is *y*(*x*), and, because latitudinal cross‐sections are circular, the lower half of the egg, below the horizontal axis, will be a mirror image. Various mathematical forms have been proposed for the meridian *y*(*x*), with parameters that can be estimated in order to match the shape of a particular egg. A general strategy is to express *y*(*x*) as a suitable modification of the equation for a circle: Preston (1953, Equation (4)) and Todd and Smart (1984, Equation (2)) proposed

(1) $$y\left(x\right)=f\left(x\right)\sqrt{1-x^{2}}.$$

Equation (4) in Preston (1953) looks different from Equation (1), but this is only because in his presentation, the longest axis of the egg is vertical, as Todd and Smart (1984) also observe. With *f*(*x*) = 1 Equation (1) gives a circle and with *f*(*x*) = *T *<* *1 it gives an ellipse with its longest axis horizontal. In the latter case, *T* is the ratio of length of the minor and major axes of the ellipse. The next simplest function, with the two parameters *T* and *a*, is *f*(*x*) = *T* (1 + *ax*), giving $y\left(x\right)=T\left(1+ax\right)\sqrt{1-x^{2}}$ which Preston called “Simple Ovoid”^2^ . Here, *T* and *a* are to be estimated for the particular egg. Smart (1969, p153) and Todd and Smart (1984, Equation (3)) both asserted that this form provides a good representation of the shape for birds’ eggs of many species but Preston (1953) did not share this opinion, preferring his three‐parameter Equation (6a) which corresponds to *f*(*x*) = *T* (1 + *ax *+ *bx* ^2^), and which he called “Standard Avian Egg‐Shape.” Both Preston (1953) and Todd and Smart (1984) note that for pyriform eggs, *f* needs to be a cubic to give a good representation of the shape. When a cubic is needed, Preston called the (pyriform) shape “Alcid Ovoid.” Preston (1953) and Todd and Smart (1984) recognized that higher order polynomials could be used in place of the cubic but comment that they found no need for this additional flexibility. Our experience is similar. Thus, the general egg shape, suitable for all bird species, is adequately represented by

(2) $$y\left(x\right)=T\left(1+ax+bx^{2}+cx^{3}\right)\sqrt{1-x^{2}}.$$

It is important to appreciate that, when the parameters *T*,* a*,* b*, and *c* are chosen to suit the particular egg, the fit is so good that for all practical purposes, these four parameters perfectly capture the shape of the egg, as the results here illustrate.

Carter (1968) proposed a two‐parameter formula which can be cast in the form of Equation (1); details can be seen in Supporting Information Section SupM1. The third parameter in that paper's title is simply the egg's length and so is unrelated to shape. Baker (2002, Equation (2)) also proposed a two‐parameter formula for egg shape, given by

(3) $$y\left(x\right)=T{\left(1+x\right)}^{1/(1+\lambda )}{\left(1-x\right)}^{\lambda /(1+\lambda )},$$

and this formula is the one used by Stoddard et al. (2017). It can be cast into the general framework provided by Equation (1) as

(4) $$y\left(x\right)=T{\left(\frac{1+x}{1-x}\right)}^{\left(1-\lambda )/(2(1+\lambda )\right)}\sqrt{1-x^{2}}.$$

Troscianko (2014, Equation (1)) offered a three‐parameter egg shape formula that becomes

(5) $$y\left(x\right)=Te^{-\alpha x-\beta x^{2}}\sqrt{1-x^{2}}$$

when cast into the general framework. More details on the derivation of Equations (4) and (5) can be found in Supporting Information Section SupM1.

Baker's, Carter's, and Troscianko's formulae have two, two, and three parameters, respectively, compared to the four in Equation (2) that were found necessary to capture the full range of egg shapes in Preston (1953) and Todd and Smart (1984). The fit of Baker's formula to pyriform eggs in particular is markedly less satisfactory than Preston's proposal with a cubic.

As formulated here, in all of these models the parameter *T* is the ratio of the diameter of the egg at the midpoint of its length (referred to as “the equatorial diameter” by Preston (1968, p457)) to the length of the egg — as can be deduced by putting *x *=* *0 in the formulae and using that the egg's length is two. Smaller values of *T* correspond to more elongated eggs.

In a somewhat different approach, Carter and Morley Jones (1970, Equation (5)) propose a formula based on polar coordinates with four parameters for shape and one for size, so it is comparable in complexity with Equation (2). They also give interpretations for their shape coefficients (calling them indices of aspect, skewness, marilynia, and platycephaly). Their suggestion does not seem to be expressible in the form of Equation (1), so details of the formulation are deferred to Supporting Information Section SupM3.

### 2.2 Fitting Preston's parameters – underlying theory

The egg image is arranged so that its longest axis is horizontal, and it is assumed here that this is the *y *=* *0 axis and that the egg has been scaled so that its poles are at *x *= −1 and *x *=* *1. Then, the coordinates of the top and bottom edge of the egg are obtained. (More details on this and on the R program for fitting, which uses EBImage (Pau, Fuchs, Sklyar, Boutros, & Huber, 2010) for image processing, are in Supporting Information Section SupM5.) The *y* values for the bottom edge are reflected in the *x*‐axis. Then, for each *x* value, *x*_*i*_, this gives two *y* values: *y*_i1_ from the top and *y*_i2_ from the bottom.

Now, the obvious model for relating the data to Equation (1) is

$$y_{\mathrm{ij}}=f\left(x_{i}\right)\sqrt{1-x_{i}^{2}}+\epsilon_{\mathrm{ij}}i=1,\ldots ,N,\;j=1,2$$

where $\epsilon_{\mathrm{ij}}$ are the errors and *N* is the number of points on each meridian. Then the error sum of squares is

$$\sum_{i=1}^{N}\sum_{j=1}^{2}{\left(y_{\mathrm{ij}}-f\left(x_{i}\right)\sqrt{1-x_{i}^{2}}\right)}^{2}.$$

Minimizing the error sum of squares is the natural way to fit the parameters to a particular egg profile: this method is used here in all cases. When *f* is a polynomial, we have a linear model — more specifically, a multiple regression without a constant term — and so standard fitting can be used, which is what Preston (1953) did.

Rather than following Preston on fitting, Todd and Smart (1984) shift attention to

$$Y_{\mathrm{ij}}=\frac{y_{\mathrm{ij}}}{\sqrt{1-x_{i}^{2}}}$$

and therefore implicitly propose the model

$$Y_{\mathrm{ij}}=\frac{y_{\mathrm{ij}}}{\sqrt{1-x_{i}^{2}}}=c_{0}+c_{1}x_{i}+c_{2}x_{i}^{2}+c_{3}x_{i}^{3}+\ldots +\frac{\epsilon_{\mathrm{ij}}}{\sqrt{1-x_{i}^{2}}}.$$

This can be fitted as a linear model by weighted least squares — although the fitting process is not addressed in Todd and Smart (1984). The weights are proportional to the inverse of the variance of the errors and so will be $\left(1-x_{i}^{2}\right)$. This fitting process is equivalent to the linear model employed by Preston. Note too that *c*_0_ is just *T* in the formulation in Equation (2). We will refer to (*c*_0_, *c*_1_, *c*_2_, *c*_3_) as Preston parameters.

To ensure the stability of the fitting process and allow high order polynomials to be used, the appropriate orthogonal polynomials are used, instead of fitting with simple powers of *x*_*i*_. These are the Ultraspherical (Gegenbaur) polynomials for weight function $\left(1-x^{2}\right)$ (see Suetin, 2002). The details of this, which involves the introduction of another parametrization for the same egg formula which has some attractive features and which yields a simple formula for the egg volume, are described in Supporting Information Section SupM2. These alternative parameters will also be referred to as Preston parameters.

### 2.3 Assessing fit

Once Preston parameters have been obtained, the egg shape they correspond to can be plotted and we call this the Preston fit. To assess the fit, the discrepancy between the Preston fit and the actual outline of the egg needs to be quantified. The measure of the quality of a fit proposed here is the square root of the average squared discrepancy between the actual egg and the fitted egg, after scaling the egg to have length one: essentially the root mean square error. This gives an error that is a length on the scale where the egg length is one. Thus, in the notation developed here, the error of a method is

(6) $$\text{error}=\sqrt{\frac{1}{8N}\sum_{i=1}^{N}\sum_{j=1}^{2}{\left(y_{\mathrm{ij}}-e_{\mathrm{ij}}\right)}^{2}}$$

where *e*_*ij*_ is the fitted value corresponding to *y*_*ij*_ and obtained from the least squares fit of the parameters, and *N* is the number of points on the egg's meridian, or equivalently the number of *x*_*i*_ values. For photographs with good resolution, *N* is large and then this formula will be an accurate representation of the discrepancy between the fitted egg and the actual egg shape. In order to compare the quality of the fit of other models with Preston's (1953) model, we need to fit them by least squares too: we indicate how this was done in Supporting Information Section SupM3.

The methods are then compared, in Sections 3.1 and 3.2 using their errors defined by Equation (6).

In fitting Equation (4), Baker (2002) and Stoddard et al. (2017) propose excluding eggs where the fit is poor. Both seem close to suggesting the square of the error defined at Equation (6) to measure the quality of the fit, but neither explain exactly how to accommodate different values of *N* and they propose slightly different exclusion rules.

### 2.4 Adding parameters to egg formulae

The beauty of Preston's proposal is that it provides an essentially exact representation for any egg shape using four parameters. It is natural to wonder whether the fits of the alternative models are improved markedly by adding parameters. It is straightforward to put additional parameters into the models of Carter (1968), Baker (2002) and Troscianko (2014). Thus, for Troscianko's formula the natural extension is

(7) $$y\left(x\right)=Te^{-\alpha x-\beta x^{2}-\gamma x^{3}}\sqrt{1-x^{2}}.$$

For Baker's model, one way to introduce the extra parameters is

(8) $$y\left(x\right)=T{\left(1+x\right)}^{1/(1+\lambda )}{\left(1-x\right)}^{\lambda /(1+\lambda )}\left(1+ax+bx^{2}\right),$$

whilst for Carter's, an analogous possibility is given in Supporting Information Equation (SEq2). In each case, this increases the number of parameters to four, giving them similar flexibility to Equation (2), so that the errors for these extensions are expected to be roughly comparable with Preston's.

### 2.5 Three shape indices

Preston's four‐parameter representation of egg shape is so good that it can replace the silhouette, allowing images to be replaced by a simple accurate summary. However, these parameters are not easily interpretable as intuitive aspects of an egg's shape. A variety of indices has been proposed that are more easily interpretable and intended to reflect aspects of shape that are considered biologically important or interesting. We first introduce three egg shape indices we refer to as Elongation, Pointedness, and Polar Asymmetry.

*Elongation* is the ratio of the length to the width at the widest point. This is not the same as 1/*T*, which uses the width at the midpoint of the egg's length (i.e. at the equator), rather than at the widest point.

*Pointedness* is the length from the point where the egg is widest to the more distant end divided by the overall length.

*Polar Asymmetry* is the ratio of the diameter of the largest circle that can fit within the egg outline and touch the egg at its blunt pole to the diameter of the largest circle within the egg outline and touching the more pointed pole.

**Figure 1:**
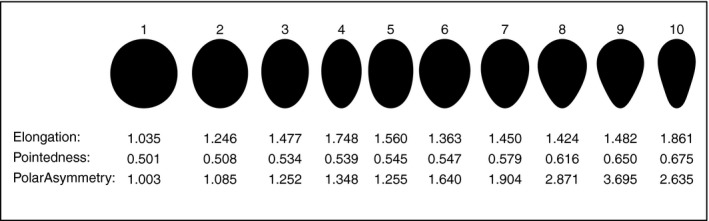
The values of the three shape indices for eggs of varied shapes. All egg images are scaled to have the same length. Key: (1) White‐breasted Kingfisher (*Halcyon smyrnensis*); (2) Adélie penguin (*Pygoscelis adeliae*); (3) Dalmatian Pelican (*Pelecanus crispus*); (4) Greater Flamingo (*Phoenicopterus roseus*); (5) Southern Brown Kiwi (*Apteryx australis*); (6) Little Grebe (*Tachybaptus ruficollis*); (7) Royal Tern (*Thalasseus maximus*); (8) King Penguin (*Aptenodytes patagonicus*); (9) Pheasant‐tailed Jacana (*Hydrophasianus chirurgus*); (10) Common Guillemot (*Uria aalge*)

Larger values of these indices correspond to greater departures from a circular shape. The values of these indices for eggs of particularly varied shapes are shown in Figure 1. For some nearly symmetrical eggs, the pole with the smaller circle (the more pointed end) can be the one that is nearer to the latitude where the egg is widest, which is the opposite of what might be expected: this is the case for egg 2 in Figure 1.

### 2.6 Other shape indices

**Figure 2:**
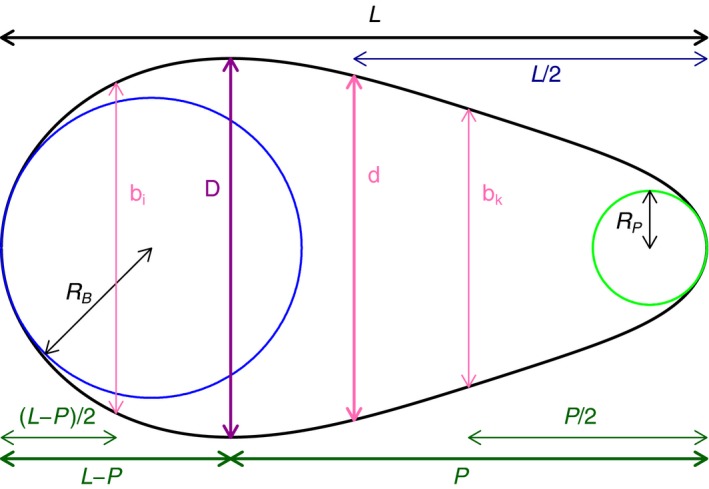
Graphical explanation of the symbols occurring in the text: L is the length of the egg; D is the largest latitudinal diameter; P is the length from the latitude of maximum diameter to the more distant pole; d is the equatorial diameter; $R_{B}$ and $R_{P}$ are the radii of the largest circles within the egg and touching the blunt and pointed pole, respectively; and $b_{i}$ and $b_{k}$ are the latitudinal diameter half way between the latitude of largest diameter and the blunt and pointed pole — $b_{i}$ is the larger of the two

We now review other indices that have been proposed. Figure 2 is a graphical representation of the symbols used in this section and Table 1 is a summary of a selection of indices.

**Table 1:** Various shape indices; symbols defined in Figure 2; “Circle” gives the value of the index for a circle

Source	Index name	Formula	Circle
*Length to breadth indices*			
Preston (1968, p456)	elongation	*D*/*L*	1
Stoddard et al. (2017, SM‐p4)	ellipticity	(*L*/*d*) − 1	0
Present study	Elongation	*L*/*D*	1
*Departure of widest latitude from equator*			
Belopol'skii (1957, p131)	Unnamed	*P*/(*L* − *P*)	1
Harris and Birkhead (1985, p174)	Shape index 1	*L*/(*L* − *P*)	2
Mänd et al. (1986, p614)	ovoidness	*P*/(*L* − *P*)	1
Deeming and Ruta (2014, p2)	asymmetry ratio	*P*/*L*	0.5
Present study	Pointedness	*P*/*L*	0.5
*Model based asymmetry*			
Stoddard et al. (2017, SM‐p4)	asymmetry	λ−1 (from eqn (4))	0
*Comparisons of the egg poles*			
Preston (1968, equation (6))	asymmetry	$\left(\sqrt{R_{B}}-\sqrt{R_{P}}\right)\sqrt{L/2}/d$	0
Preston (1968, equation (7))	bicone	$\left(\left(\sqrt{R_{B}}+\sqrt{R_{P}}\right)\sqrt{L/2}/d\right)-1$	0
Preston (1968, equation (10))	Asymmetry	$\left(R_{B}-R_{P}\right)L/D^{2}$	0
Preston (1968, equation (11))	Bicone	$\left(\left(R_{B}+R_{P}\right)L/D^{2}\right)-1$	0
Mityay et al. (2015, p93)	asymmetry	$R_{P}/R_{B}$	1
Mytiai and Matsyura (2017, p265)	asymmetry	$(R_{B}-R_{P})/D$	0
	infundibular	$R_{B}/D$	1
	cloacal	$R_{P}/D$	1
	interpolar	$\left(L-(R_{B}+R_{P})\right)/D$	0
	complementarity	$\frac{\left(1+R_{B}/L\right)\left(1+R_{P}/L\right)}{1-R_{B}/L-R_{P}/L}$	∞
Present study	Polar Asymmetry	$R_{B}/R_{P}$	1
*Comparisons using intermediate latitudes*			
Mänd et al. (1986, p614)	pear‐shapedness	$\left(b_{i}-b_{k}\right)/b_{i}$	0
	conidity	$(b_{i}-b_{k})/D$	0
	blunt‐end convexity	$(2b_{i}/D)-1$	$\sqrt{3}-1$
	sharp‐end convexity	(2*b*_*k*_/*D*) − 1	$\sqrt{3}-1$
*Using egg volume V*			
Mänd et al. (1986, p614)	plumpness	3*V*/(4*πLD* ^2^)	1

#### 2.6.1 Length to breadth indices

The first, and most obvious, index is what is called elongation (by, for example, example, Preston, 1968, p456): the ratio of the length of the largest latitudinal diameter (*D*) – often simply called its maximum diameter or breadth – to the length (L) of the egg. In this study, Elongation is defined as the reciprocal of elongation, so that its values are always ≥1, and larger values correspond to more elongation.

As an alternative to elongation, the ratio of the equatorial diameter (*d*) to the length of the egg could be used: *d*/*L*. This is the parameter *T* in the formulae in Section 2.1. It is ≤1 and is one for a circle. Thus, 1/*T* is ≥1, with larger values corresponding to less and less spherical eggs and 1/*T* agrees with Elongation for eggs which have their maximum diameter at their equator. Stoddard et al. (2017) use this index, with *T* obtained via Equation (4), but subtract one from it to make zero correspond to a circle. Thus, their index, which they call ellipticity, is (1/*T*) − 1, which in terms of direct egg measurements is (*L*/*d*) − 1 and in Preston parameters is (1/*c*_0_) − 1.

#### 2.6.2 Departure of widest latitude from equator

In asymmetric eggs, the latitude of the maximum diameter will be displaced from the equator. That leads naturally to seeking a second index based on this displacement. In this study, we use Pointedness: the length from the latitude of maximum diameter to the more distant pole (*P*) divided by the overall length (*L*). Similar indices have been used by Belopol'skii (1957), Harris and Birkhead (1985) and Mänd et al. (1986). Their proposals are all monotonic transformations of Pointedness and so are equivalent to it (in that they will have a perfect Spearman correlation with Pointedness). The same index, called the asymmetry ratio, has been proposed by Deeming and Ruta (2014, p2)^3^ where “equatorial axis” is the latitude of maximum diameter (which is not the sense of “equatorial” here) — so their definition is indeed identical to that of Pointedness.

#### 2.6.3 Model based asymmetry

Stoddard et al. (2017) define their asymmetry index to be λ−1 having fitted the formula (4) to the egg profile. The −1 is to make the value of the index zero for a circle. (In fact, they use max{λ,1/λ}−1 to deal properly with nearly symmetrical cases, but this is a minor refinement.) The main difficulty with this index is that the model in Equation (4) (i.e. Baker, 2002) does not fit well in all cases (see Section 3).

#### 2.6.4 Comparisons of the egg's poles

A variety of proposals exist for indices based on the curvature of the poles of the egg. Preston (1968, p456) notes that even for symmetrical eggs, the two ends can be more or less pointed: “both ends may be conspicuously pointed as in the tinamous, or they may both be conspicuously blunt as in the hummingbirds.” Thus, he sought an index that could reflect this difference, which he called bicone. In addition, and less subtly, there can be asymmetry, with the curvature of the two poles being markedly different. Based on this thinking and his modeling Preston (1968, Equation (6), Equation (7)) proposed two indices, bicone and asymmetry, derived from the curvature at the poles. He made various approximations and simplifications to derive alternative indices (Preston, 1968, Equation (10), Equation (11)), which he calls Bicone and Asymmetry, that were easier to obtain through field measurements, although as mentioned above, these entailed the use of a spherometer. Now that photographs can be more easily analyzed, finding the largest circle within the egg and touching its pole provides a sensible alternative to using a spherometer.

In order to describe the indices based on the curvature at the poles, we follow Preston's (1968) terminology: let *R*_B_ and *R*_P_ be the radii of the largest circle at the blunt and the pointed end, respectively, as illustrated in Figure 2. It is now straightforward to obtain versions of Preston's indices, either in their original form or in his operational substitutions. Although their approach is rather different, Mityay et al. (2015), Mityay, Strigunov, and Matsyura (2016) and Mytiai and Matsyura (2017) also suggest a variety of indices based on the radii of circles. They are not fully consistent in naming these nor in the formulae. In particular, the “index of asymmetry” in Mityay et al. (2015) is *R*_P_/*R*_B_, the reciprocal of Polar Asymmetry, but the “index of asymmetry” in Mytiai and Matsyura (2017) is different: it is (*R*_B_ − *R*_P_)/*D*. A selection of indices from Mytiai and Matsyura (2017) is included in Table 1.

#### 2.6.5 Comparisons using intermediate latitudes

Instead of using the curvature of the poles, Mänd et al. (1986) define indices^4^ based on the diameters for the latitudes midway between the latitude of largest diameter and the two poles. Let *b*_*i*_ and *b*_*k*_ be these two latitudinal diameters, with *b*_*i*_ being the larger of the two. In Mänd et al. (1986, Figure 3) and in Figure 2, the larger diameter, *b*_*i*_, is obtained from the latitude nearer to the blunt pole, and this is typical. However, in nearly symmetrical eggs the larger diameter can be nearer the more pointed pole, a possibility which Mänd et al. (1986) may not have envisaged. The definition used in this study makes *b*_*i*_ the larger of the two intermediate diameters even in these cases.

**Figure 3:**
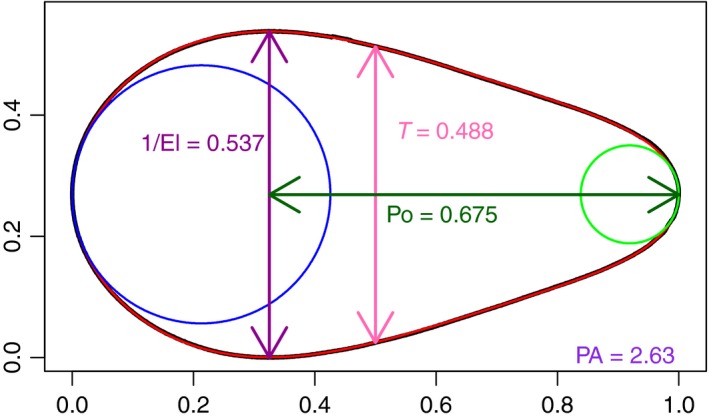
The actual egg shape of C126 is the black outline; the Preston fit is in red. The error, as defined at Equation (6), is 0.00091. The length of the egg has been scaled to be one. The two circles are the largest possible that touch the end of the egg and are wholly within the (Preston fit to the) egg. Then, the Polar Asymmetry (PA) is the ratio of the diameter of the larger (blue) to the smaller (green) circle. Po is the pointedness. El is the Elongation. *T* is the equatorial diameter

Mänd et al.’s (1986) pear‐shapedness and conidity are based on the difference in these two distances, so will both be zero for any symmetrical egg. For an asymmetrical egg, *b*_*k*_ will be less than *b*_*i*_, with larger values of these indices corresponding to greater asymmetry. The other two indices, blunt‐end convexity and sharp‐end convexity, seek to measure the pointedness of each end separately and so are similar in spirit to Mytiai and Matsyura's (2017) infundibular and cloacal.

#### 2.6.6 Using egg volume

Mänd et al. (1986) propose an index which compares the egg volume, *V*, to that of a prolate ellipsoid (i.e. one with circular cross‐sections on the minor axis). They proposed 400*V*/(*πLD* ^2^). In Table 1, the multiplier has been adjusted to give an index value of one if the egg shape was an ellipse with major axis *L* and minor axis *D*.

#### 2.6.7 Scaling and centering of indices

A shape index is, necessarily, independent of size and so has no length scale. By considering the value that the index will take for a circle, the index can be rescaled so that a circle gives a value of one or re‐centered to make the value for a circle zero. For example, Stoddard et al. (2017) subtracted one from 1/*T* and from λ to make the value for a circle zero and Preston (1968) subtracts one in the definition of Bicone for the same reason. Such maneuvers make no essential difference but do lead to some of the differences in naming and definitions. Here, the scaling of Mänd et al.’s (1986) pear‐shapedness and conidity has been adjusted: the originals were 100 times the formulae in Table 1.

#### 2.6.8 Data driven index‐like summaries

Once egg profiles are in a standard orientation (which here is horizontal, with the *x*‐axis along the longest axis) and size (which here is the maximum length standardized to be two), a collection of coordinates on the profile taken at a fixed collection of *x*‐values is a multivariate observation on an egg profile. As such, techniques like principal components can be used to explore and summarize shape. This is, in essence, the approach used in Deeming and Ruta (2014) and Deeming (2017). In particular, Deeming and Ruta (2014) perform a principal component analysis on a wide range of egg shapes and observe that the first principal component is highly correlated with elongation and the second with their asymmetry ratio and that the first two components account for 89.48% and 7.96%, respectively, of the total variance, confirming that these two indices account for much of the variation in egg shape; Deeming (2017) explores the relationship of various factors on these principal components over a large selection of bird species.

**Figure 4:**
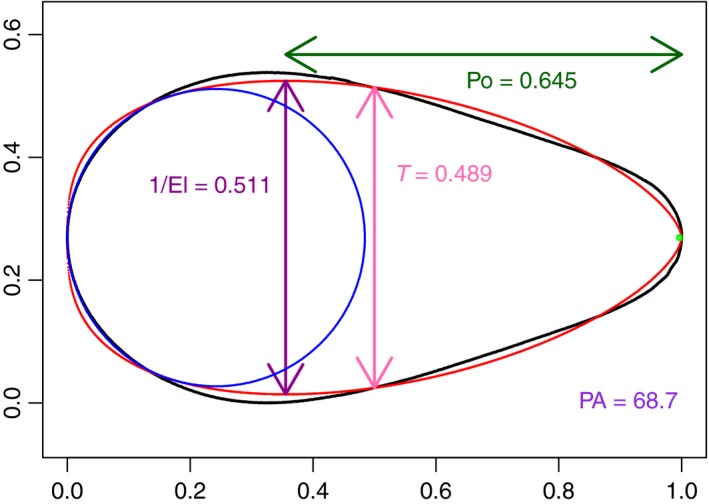
The actual egg shape of C126 is the black outline; the Baker fit is in red. The error, as defined at Equation (6), is 0.0116. The values of Po, 1/El, and *T* based on the Baker fit are indicated. The Polar Asymmetry (PA) is very large because of the very small circle at the more pointed end

**Figure 5:**
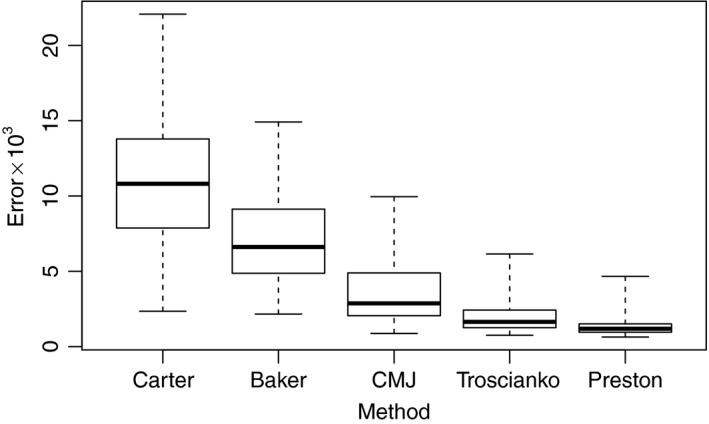
Boxplots comparing the error defined at Equation (6) (multiplied by 1,000) for the methods of Carter (1968), given in Supporting Information Equation (SEq1), the methods of Baker (2002) and Troscianko (2014), given in Equations (4) and (5), the method of Carter and Morley Jones (1970), described in Supporting Information Equation (SEq6), labeled CMJ, and the method of Preston (1953) given in Equation (2). The results are for 132 eggs of various species: 18 *Uria aalge*, 16 *Uria lomvia*, 7 *Alca torda*, 11 *Aptenodytes patagonicus*, 10 *Lanius collurio*, 10 *Phalacrocorax carbo*, 10 *Gallus gallus domesticus*, 10 *Spheniscus humboldti*, 10 *Eudyptes pachyrhynchus*, 30 *Larus fuscus*. The heavy line is the median, the boxes extend between the upper and lower quartiles, the whiskers extend to the minimum and maximum

### 2.7 Egg characteristics from Preston parameters

Figure 2 is a graphical representation of the measurements that are used to define various indices. All of these can be obtained from the Preston parameters: the two radii, *R*_B_ and *R*_P_, are the most complicated to obtain, but are easily found by a suitable search procedure. Furthermore, assuming circular cross‐sections, the formula for egg shape can also be used to find other egg characteristics: for example, the surface area, volume, or “contact index” (as in Birkhead, Thompson, Jackson, et al., 2017), which indicates how much of an egg resting naturally makes contact with the substrate. In particular, Supporting Information Equation (SEq4) shows how to obtain the egg volume from the Preston parameters. It is also straightforward to fit the alternative models, for example that in Equation (4), to the Preston fit for the egg, instead of going back to the original photograph. As the Preston fit is so good, this produces parameters very similar to those obtained from fitting to the photograph directly, as is illustrated in Supporting Information Section SupM4. Thus, the various indices in Table 1 can be readily obtained from the Preston parameters. In a similar vein, the approach in Deeming and Ruta (2014) can be applied to the shapes obtained from the Preston parameters of a collection of eggs, rather than to the original photographs.

## 3 RESULTS

### 3.1 Comparisons using a pyriform egg

The focus here is on the avian egg shape that historically has been the most challenging: pyriform. A guillemot egg (labeled C126, see Supporting Information Figure SF10) was selected, because of its marked pyriform shape, to use as a test case for the various formulae. In Figure 3, the Preston fit is superimposed on the egg outline: both are plotted “thinly,” so that the close fit is clear. The egg outline is drawn using the $\left(x_{i},y_{\mathrm{ij}}\right)$ pairs introduced at the start of Section 2.2: there are *N *=* *3,488 points on each meridian, so the egg outline based on them is, for practical purposes, exact. The error, as defined at Equation (6), is 0.00091. Various derived quantities (Elongation, Pointedness, Polar Asymmetry, and equatorial diameter) are also marked on the figure.

In Figure 4, the Baker fit is illustrated for the same egg. The fit is poor (the error, as defined at Equation (6), is 0.0116) and, if the Baker fit were used to estimate our derived quantities, several of them would be in error. As can be seen from Figure 4, Polar Asymmetry would be vastly overestimated, because of the excessively pointed end in the Baker fit. Elongation would be overestimated and Pointedness would be underestimated. For this egg, it looks as though the equatorial diameter would be accurately estimated through the Baker fit. Baker (2002) proposed omitting eggs like this, where the fit is poor, and that recommendation is followed by Stoddard et al. (2017, SM‐p4, Figures S2 and S8.A). This is a serious drawback when applying the method to draw conclusions about the full range of avian egg shapes.

Comparing the fit in Figures 3 and 4, the error (given by Equation (6)) for the Baker fit (i.e. fitting Equation (4)) is more than 12 times that of the Preston fit. For comparison, the error for the Troscianko fit (i.e. fitting Equation (5) – illustrated graphically in Supporting Information Figure SF11) is five times that of the Preston error, and the error for the Carter fit (i.e. fitting Supporting Information Equation (SEq1)) is 17 times that of the Preston error. For egg C126, the four‐parameter extensions in Equations (7), (8) and Supporting Information Equation (SEq2) give errors that are, respectively, 1.8, 2.7, and 3.4 times the Preston error. These last three all correspond to good fits “by eye,” as is illustrated in Supporting Information Figure SF12 for the one with the largest error (i.e. Supporting Information Equation (SEq1)) but they are still slightly poorer than the Preston fit. The method proposed by Carter and Morley Jones (1970) produces 10.7 times the Preston error and the fitted egg has visible undulations, illustrated in Supporting Information Figure SF13, and so does not accurately capture this egg's shape.

### 3.2 Comparisons of fit over a selection of eggs

Figure 5 gives the errors of each method over a selection of 132 eggs from ten species. It shows that the errors from Preston's method are generally smaller than those of the others. The actual Preston errors range from 0.00064 to 0.00466, based on values of *N* that range from 1706 to 3622. Additional comparisons are included in Supporting Information Section SupM6. These show that when the proposals of Carter (1968), Baker (2002) and Troscianko (2014) are augmented to each have four parameters, as in Equations Supporting Information (SEq2), (8) and (7), respectively, they provide fits of comparable quality to Preston's.

For a circular egg profile, all methods work well, so Figures 5 and Supporting Information Figure SF2 cannot, and are not intended to, show that the difference in quality of the fit is important in all cases. Rather, they demonstrate that Preston's method is satisfactory for all eggs, including those where the alternatives proposed elsewhere work less well. Preston's method is the best choice for providing a consistently accurate summary over a range of egg shapes.

### 3.3 The importance of egg position

The validity of the Preston summary relies on the egg being horizontal (i.e. the line through the poles being horizontal) when photographed. Otherwise, for example, the assumption of circular cross‐section will be invalid and so using the Preston summary to obtain an egg volume will give an incorrect answer. Most birds’ eggs do not rest naturally in a horizontal position. A pointed egg that is at rest will have its pointed end lower and its blunt end higher than would be the case if it were horizontal. Thus, the length will be foreshortened and so will be shortened when compared to the breadth. To explore the kind of biases this will introduce, data on 185 eggs of various species that were photographed in both the horizontal and in their resting position are compared in Supporting Information Section SupM7. It is clear from those results that marked biases are introduced if eggs in a resting position are used.

### 3.4 Comparison of indices

For the three indices introduced here in Section 2.5, Elongation, Pointedness, and Polar Asymmetry, an interactive 3d‐plot (Supplementary‐Material2.html, see Data Accessibility) of their values on a large collection of eggs illustrates that, for pyriform eggs, each contains information not in the other two, as the cloud has marked scatter, regardless of the angle it is viewed from.

Preliminary observations on the relationships between various indices in Table 1 are in Supporting Information Section SupM8. The strength and form of these relationships will depend on the the collection of eggs used. As the main focus is dealing satisfactory with pyriform eggs, the main data used to compare the indices are on 735 *Uria aalge* eggs.

The shape of the correlation matrix in Supporting Information Figure SF7 shows four groups of indices, indicated by the high correlations near the diagonal. We identify indices that typify these groups. The first corresponds to Elongation, the second to Preston's (1968) bicone, the third to Pointedness, and the fourth to Polar Asymmetry. Thus, for the complexities of pyriform shape, just as four parameters are needed for the Preston fit, four shape indices capture different aspects of their shape. Of these four, Preston's (1968) bicone is rather different from the other three, in that it is an index of the average curvature at the two poles, and seems less directly related to the main features of the shape. The other three provide a satisfactory basis for comparisons of pointedness in a general sense.

## 4 DISCUSSION

The demonstrated merits of Preston's approach to summarizing egg shape make it a proper starting point for all future studies that aim to capture egg shape closely. Using it as the basis of quantifying egg shape would allow the sorts of comparative study pioneered by Stoddard et al. (2017) to be conducted with rather more confidence.

The accuracy of the shape obtained means that the Preston parameters can be used to compute any desired biologically sensible indices without recourse to the original egg or its photograph. As noted already, other methods can provide an adequate summary of some eggs, but four parameters (as in Preston's method) are needed to be assured of a good summary of all eggs. Even if the fit of a method is not as good as Preston's, it may well be satisfactory for deriving with reasonable accuracy some egg characteristics. There will, for example, be only relatively minor differences in the estimate of egg volume based on different methods. However, in contrast, Polar Asymmetry is an example of an index where the parametric shape needs to mimic the shape of the actual egg closely at each pole to obtain an accurate estimate (c.f. Figures 3 and 4 and Supporting Information Figure SF11).

Given the effectiveness of Preston's approach, a database summarizing, through Preston parameters, a large collection of appropriately taken photographs of eggs would be a valuable resource for future research.

Errors of asymmetry and surface imperfections are incorporated into the error in the fitting. Thus, the consistently small errors found here for the four‐parameter models (see Supporting Information Figure SF1: maximum 0.005, three‐quarters below 0.002, where the egg has length one) indicate that these aspects are genuinely minor. There is a case for regarding a good smooth fit (like Preston's) to the egg shape as being its “real” shape, with biological significance, with minor imperfections being genuinely insignificant randomness.

The quality of the Preston fit means that the way the photographs are taken and the processing of the images are important. The method of taking photographs and the adjustment for lens distortion are described in Birkhead, Thompson, and Biggins (2017, Supplementary Material).

The three indices Elongation, Pointedness, and Polar Asymmetry each measure aspects of egg shape in an intuitive way. For pyriform eggs, the results show that each of these indices contributes information about the egg's shape that the other two do not. Of the other indices that have been proposed, none is clearly more suitable based on their correlations and the immediacy of interpretation. An extensive comparative study of the range of indices proposed across a full range of egg shapes would be needed to establish fully their relative merits, their commonalities, and their effectiveness at capturing biologically interesting aspects of shape.

## 5 CONCLUSION

We demonstrate that the method proposed by Preston (1953), and revisited in Preston (1968) and Todd and Smart (1984), works accurately for all egg shapes and is better than the existing alternatives. The programs supplied provide a straightforward way to obtain the Preston parameters for a collection of suitable photographs and illustrate how to use these parameters to derive other egg characteristics. To use these methods, it is important that the photographs are of eggs positioned horizontally, otherwise biases are introduced. The present study establishes the value of using all three of the indices Elongation, Pointedness, and Polar Asymmetry when pyriform eggs are being considered.

## AUTHORS’ CONTRIBUTIONS

TRB conceived the study; JDB and TRB wrote the paper; JET and TRB measured and photographed the eggs; and JDB did the mathematics and the programming.

## DATA ACCESSIBILITY

The R programs described in Supporting Information Section SupM5 and suitable test data, an R script that generates all the analyses in this paper and the data sets used in the analyses, and the file Supplementary‐Material2.html are available through Dryad, https://doi.org/10.5061/dryad.8kv2b20.

## ENDNOTES

1 Sections in the document Supplementary Material, provided as Supporting Information, have the prefix SupM, figures have the prefix SF and equation numbers have the prefix SEq.

2 It is worth nothing that Equation (4.1) in Okabe (1952) is “Simple Ovoid”: Consequently, his approach to the bio‐mechanical understanding of egg formation is automatically constrained to produce egg shapes in this class.

3
A slightly different definition occurs in Deeming and Ruta (2014, figure 5, p9), but that one is incorrect (C. Deeming, pers com, January 2018).

4 Mänd et al. (1986) mention that some of their indices were derived from those of Kostin (1977).
