# Supplementary material for: Accurately quantifying the shape of birds’ eggs
Source: Ecol Evol. 2018 Sep 5;8(19):9728–38. doi: 10.1002/ece3.4412 (PMC6202712; doi:10.1002/ece3.4412)
Supplement: Supplementary file 1 [file ECE3-8-9728-s001.pdf]

Supplementary Material

SupM1 Expressing models in the general form

This section gives more detail on how various proposed formulae for egg shape are cast into the general form (1).

Baker (2002)

Starting from Baker (2002, eq(2)) and using that $(1-x^2) = (1+x)(1-x)$,

$$\begin{aligned}
T(1+x)^{1/(1+\lambda)}(1-x)^{\lambda/(1+\lambda)} &= T(1+x)^{1/(1+\lambda)}(1-x)^{\lambda/(1+\lambda)}\frac{(1-x^2)^{1/2}}{(1+x)^{1/2}(1-x)^{1/2}} \\
&= T(1+x)^{1/(1+\lambda)-1/2}(1-x)^{\lambda/(1+\lambda)-1/2}(1-x^2)^{1/2} \\
&= T(1+x)^{(1-\lambda)/(2(1+\lambda))}(1-x)^{-(1-\lambda)/(2(1+\lambda))}\sqrt{(1-x^2)} \\
&= T\left(\frac{1+x}{1-x}\right)^{(1-\lambda)/(2(1+\lambda))}\sqrt{(1-x^2)},
\end{aligned}$$

which is the right hand side of equation (4).

Carter (1968)

Carter (1968, eq(3)) proposes the following formula for the meridian, with poles at 0 and 1,

$$y = b\left[\frac{1}{4} - \left(X^p - \frac{1}{2}\right)^2\right]^{1/2} \quad \text{for } 0 \le X \le 1.$$

Letting $X = (x+1)/2$ moves the poles to -1 and 1, giving, for $-1 \le x \le 1$

$$\begin{aligned}
y &= b\left[\frac{1}{4} - \left(\left(\frac{x+1}{2}\right)^p - \frac{1}{2}\right)^2\right]^{1/2} \\
&= b\left[\frac{1}{4} - \left(\left(\frac{x+1}{2}\right)^{2p} - \left(\frac{x+1}{2}\right)^p + \frac{1}{4}\right)\right]^{1/2} \\
&= b\left[\left(\frac{x+1}{2}\right)^p - \left(\frac{x+1}{2}\right)^{2p}\right]^{1/2} \\
&= b\left[\left(\frac{x+1}{2}\right)^p \left(1 - \left(\frac{x+1}{2}\right)^p\right)\right]^{1/2}.
\end{aligned}$$

The equatorial diameter, T, is the value of y when $x = 0$; thus

$$T = b\left(\frac{1}{2}\right)^p\left(1-\left(\frac{1}{2}\right)^p\right).$$

Using this to substitute for b now gives

$$\begin{aligned} y &= \frac{T}{\left(\frac{1}{2}\right)^p\left(1-\left(\frac{1}{2}\right)^p\right)}\left[\left(\frac{x+1}{2}\right)^p\left(1-\left(\frac{x+1}{2}\right)^p\right)\right]^{1/2} \\ &= \frac{T}{\left(\frac{1}{2}\right)^p\left(1-\left(\frac{1}{2}\right)^p\right)}\left[\frac{1}{(1-x^2)}\left(\frac{x+1}{2}\right)^p\left(1-\left(\frac{x+1}{2}\right)^p\right)\right]^{1/2}\sqrt{1-x^2} \end{aligned}$$

which rearranges to give

$$y(x) = T\left(\frac{1}{(1-x^2)}\frac{\left(\frac{x+1}{2}\right)^p}{\left(\frac{1}{2}\right)^p}\frac{\left(1-\left(\frac{x+1}{2}\right)^p\right)}{\left(1-\left(\frac{1}{2}\right)^p\right)}\right)^{1/2}\sqrt{1-x^2}, \tag{SEq1}$$

with parameters T and p.

Equation (SEq1) can be extended to have four parameters, after the fashion of (8), as

$$y(x) = T\left(\frac{1}{(1-x^2)}\frac{\left(\frac{x+1}{2}\right)^p}{\left(\frac{1}{2}\right)^p}\frac{\left(1-\left(\frac{x+1}{2}\right)^p\right)}{\left(1-\left(\frac{1}{2}\right)^p\right)}\right)^{1/2}(1+ax+bx^2)\sqrt{1-x^2}. \tag{SEq2}$$

Troscianko (2014)

Troscianko (2014, eg(1)) offered the following egg shape formula that assumes the poles of the egg are at $l = 0$ and $l = 1$,

$$y = \frac{ae^{-l^2/(2b^2)+cl/b^2-c^2/(2b^2)}}{\pi b}\sqrt{1-l}\sqrt{l} \quad \text{for } 0 \le l \le 1.$$

Letting $l = (x+1)/2$ moves the poles to -1 and 1, giving, for $-1 \le x \le 1$

$$y = \frac{ae^{-((x+1)/2)^2/(2b^2)+c((x+1)/2)/b^2-c^2/(2b^2)}}{\pi b}\sqrt{\frac{1-(x+1)}{2}}\sqrt{\frac{(x+1)}{2}}. \tag{SEq3}$$

Expanding the exponent here,

$$\begin{aligned} -\frac{((x+1)/2)^2}{2b^2}+\frac{c(x+1)/2}{b^2}-\frac{c^2}{2b^2} &= -\frac{x^2+2x+1}{8b^2}+\frac{cx+c}{2b^2}-\frac{c^2}{2b^2} \\ &= -\frac{x^2}{8b^2}-\frac{2x}{8b^2}-\frac{1}{8b^2}+\frac{cx}{2b^2}+\frac{c}{2b^2}-\frac{c^2}{2b^2} \\ &= -\frac{1}{8b^2}x^2-\left(\frac{1}{4b^2}-\frac{c}{2b^2}\right)x+\left(-\frac{1}{8b^2}+\frac{c}{2b^2}-\frac{c^2}{2b^2}\right) \\ &= -\beta x^2-\alpha x+K \end{aligned}$$

for suitable β, α, and K. Also

$$\sqrt{\frac{1-(x+1)}{2}}\sqrt{\frac{(x+1)}{2}} = \frac{\sqrt{1-x^2}}{2}.$$

Substituting these into (SEq3), gives

$$y = \frac{a}{2\pi b} e^{-\alpha x - \beta x^2 + K} \sqrt{1-x^2}$$

and setting $T = ae^K/(2\pi b)$ gives (5).

SupM2 Fitting Preston parameters – further theory

We first define the Ultraspherical (Gegenbaur) polynomials for weight function $(1-x^2)$ (see Suetin (2002) with $\lambda = 3/2$), which are the appropriate orthogonal polynomials here. Unnormalised versions of the polynomials can be obtained recursively as

$$C_0(x) = 1,$$
$$C_1(x) = 2\lambda x,$$
$$C_n(x) = \frac{1}{n}\left(2x\left(n+\lambda-1\right)C_{n-1}(x) - (n+2\lambda-2)C_{n-2}(x)\right) \qquad (n \geq 2).$$

These are then normalized using

$$N_n = \frac{3}{4}\frac{\pi 2^{1-2\lambda}\Gamma(n+2\lambda)}{\Gamma(n+1)(n+\lambda)\Gamma(\lambda)^2}$$

to give

$$G_n(x) = \frac{C_n(x)}{\sqrt{N_n}}.$$

With this normalization,

$$\int_{-1}^{1} G_n(x)^2(1-x^2)dx = \frac{4}{3}.$$

Then the linear model with a polynomial of order J, for Y_{ij} defined in §2.2, becomes

$$Y_{ij} = \sum_{j=0}^{J} M_j G_j(x_i) + \frac{\epsilon_{ij}}{\sqrt{1-x_i^2}},$$

with the M_j being coefficients to be estimated. The M's will also be called Preston parameters, along with the c's. Taking $J = 3$ gives a model equivalent to (2).

The first advantage of using orthogonal polynomials is that the least squares estimates of the lower order terms are unchanged when higher order terms are dropped. This circumvents the difficulty mentioned by Preston (1953, p176) that arises with the estimation of the actual coefficients of the powers of x.

Given the fit

$$y(x) = \sum_{j=0}^{J} M_j G_j(x)\sqrt{1-x^2}$$

and assuming circular cross sections, the volume of the egg (in units where the total length of the egg is 2, since the long axis of the egg runs from -1 to 1) is

$$\begin{aligned}\int_{-1}^{1} \pi y(x)^2 dx &= \pi \int_{-1}^{1} \left(\sum_{j=0}^{J} M_j G_j(x)\right)^2 (1-x^2)dx \\ &= \pi \int_{-1}^{1} \sum_{j=0}^{J} M_j^2 G_j(x)^2 (1-x^2)dx \\ &= \frac{4\pi}{3} \sum_{j=0}^{J} M_j^2,\end{aligned}$$

where the second equality uses orthogonality and the third uses the normalization. If the overall length of the egg is L, then the volume estimate is

$$\text{egg volume } = \left(\frac{L}{2}\right)^3 \frac{4\pi}{3} \sum_{j=0}^{J} M_j^2. \quad \text{(SEq4)}$$

This provides a natural interpretation of the successive fits using the orthogonal polynomials: with $J = 0$, the volume is that of the best ellipsoid, with $J = 1$, that of the best simple ovoid, and so on. This simple interpretation in terms of approximating volumes is the second attractive feature of the parametrisation based on the orthogonal polynomials.

Focussing on the cubic case, the two representations

$$\sum_{j=0}^{3} c_j x^j \quad \text{and} \quad \sum_{j=0}^{3} M_j G_j(x)$$

are completely equivalent. It is therefore routine algebra to obtain formulae for the c's in terms of the M's. They are:

$$c_0 = M_0 - \frac{1}{2}\sqrt{\frac{7}{2}}M_2; \;\; c_1 = \sqrt{5}M_1 - \frac{15}{2}\sqrt{\frac{3}{10}}M_3; \;\; c_2 = \frac{5}{2}\sqrt{\frac{7}{2}}M_2; \;\; c_3 = \frac{35}{2}\sqrt{\frac{3}{10}}M_3. \quad \text{(SEq5)}$$

Consequently, a fit obtained using the M's can be converted directly to the equivalent one using c's.

SupM3 Fitting other models

To compare their performance with Preston's (1953) model we need to fit the other models mentioned by minimising their error sum of squares. Here we describe briefly how this was done.

There are some additional complications in fitting the models proposed by Carter (1968), Baker (2002) and Troscianko (2014). Specifically, as can be seen from equations (SEq1), (4) and (5), these models are not linear in the parameters, which means that they require plausible initial values for the parameters to start the fitting process. The fitting can then be done in R, using (`nls`). We have successfully fitted these models to the data from photographs described in §2.2 and §SupM5, using Preston's fit to specify the initial values for the parameter estimates for the iterative fitting process. Fitting (SEq2), (7) and (8) presented some additional difficulties with convergence, which were circumvented by expressing the parametrizations somewhat differently.

The formula for the egg profile in Carter & Morley Jones (1970, eq(5)) is in polar coordinates (r, θ), with the origin for x at the widest point of the egg. As they note, the egg length is $2(K_1 + K_2)$ (as is seen by setting $\theta = 0$ and $\theta = \pi/2$) and so the standardization to egg length being two, used here, entails $K_1 = 1 - K_2$. Incorporating this, and multiplying eq(5) in Carter & Morley Jones (1970) by $\sin(\theta)$ throughout gives a formula for the y-coordinate of the egg*:

$$y = \Big(1 + K_2(\cos(2\theta) - 1) - K_3\cos^3(\theta) - K_4\sin^2(2\theta) + K_5\sin^3(2\theta)\Big)\sin(\theta). \qquad \text{(SEq6)}$$

Given the data x_i and the corresponding y_{i1} and y_{i2} introduced at the start of §2.2, let x^* be the average of the x values where $(y_{i1} + y_{i2})$ is a maximum, so that this is the place where the egg has maximum diameter. Define θ_{ij} to be the angle $(x_i - x^*, y_{ij})$ makes with the x-axis (`atan2(x,y)` in R). Then the following relationship

$$y_{ij} \approx \Big(1 + K_2(\cos(2\theta_{ij}) - 1) - K_3\cos^3(\theta_{ij}) - K_4\sin^2(2\theta_{ij}) + K_5\sin^3(2\theta_{ij})\Big)\sin(\theta_{ij})$$

*Carter & Morley Jones's (1970) formula is for $y \geq 0$, and so $0 \leq \theta \leq \pi$: the presence of the $\sin^3(2\theta)$ term means that the formula does not give a symmetrical shape when extended to $\pi < \theta \leq 2\pi$.

can be fitted by standard least squares (treating it as a multiple regression) to find K_2, K_3, K_4 and K_5. Instead of a regression on y, Carter & Morley Jones (1970) propose a regression on r, which can be done in the same way. We found regression on r produced similar answers, but with marginally greater error.

SupM4 Baker parameters from the Preston fit

Given Preston parameters (c_0, c_1, c_2, c_3) the values along the fitted meridian can be calculated at equi-spaced points. Values of x ranging from -0.9999 to $+0.9999$ in steps of 0.0001 were used. Then, Baker's model (equation (4)) was fitted using `nls`, as in §SupM3. Baker parameters were obtained by this method for the 132 eggs used in the comparison of the fits of various methods in Fig. SF2. The mean difference between T obtained directly and that obtained from the Preston fits was -0.0000051 with a range from -0.0000696 to 0.0000519. The mean difference between λ obtained directly and that obtained from the Preston fits was 0.000152 with a range from -0.0036913 to 0.0034385. Thus, no important errors will be introduced by using the Preston parameters to obtain Stoddard et al.'s (2017) ellipticity and asymmetry rather than obtaining them directly photographs.

SupM5 Egg shape analysis – the R program

Here the approach to the analysis in R will be described. Further documentation is included in the comments within the program (`Preston.R`) and a brief users guide. The main program runs on a collection of images of eggs, where in each image the egg is the largest object in the picture. The image processing uses the package `EBImage`, (Pau et al. 2010). To record the shape of the egg accurately it needs to be positioned with the axis through the poles horizontal when photographed (otherwise bias and additional noise are introduced: see §SupM7). To obtain a clear outline, the eggs should be lit from behind as on a light box.

The default is that each egg image is in a `jpg` file with the filename of the form

eggname.jpg. In addition, an input file in csv format must contain at least three columns and those three must have names eggname, maxlength, maxbreadth. These are the name of the egg, which is the prefix of the image filename, and the maximum length of the egg and its maximum breadth (L and D in Fig. 2) in mm. It may contain other columns of supplementary information which will be passed through to the output. Optionally there can be column called light.background with values TRUE or FALSE, depending on whether the background is lighter than the egg or not; if this column is not provided the value TRUE is assumed. Photographs on a dark background work less well, unless the egg itself is uniformly bright. Optionally there can also be a column called image.suffix with values jpg, png, or tif giving the suffix for that egg's image file. If the column is not provided jpg format is assumed: the other formats may produce warnings in the processing.

The actual measurements provided (in the columns maxlength and maxbreadth) are not needed to obtain shape indices, since these are independent of actual lengths, but the length is needed if actual measurements are to be derived (such as the volume). The actual measurements are used in a consistency check on the attributes extracted from the egg image. To do this their ratio is compared with the corresponding ratio from the photograph. A discrepancy indicates either a measurement error or an egg that was not horizontal when photographed. The program flags eggs where the discrepancy exceeds 2%.

Using EBImage, each photograph is read, converted to a black and white image, with white as the foreground, and processed to identify and keep only the largest white object (the egg). Then EBImage is used to rotate the image so that the longest axis of the egg is horizontal. The image is stored as a rectangular matrix, with 0/1 entries for each pixel. Thus the top and bottom edges of the egg can be found as the maximum and minimum places, for each pixel x value, that have a one in the matrix. This identifies x_i and the corresponding pair $y_{ij},\ j = 1, 2$. Then least squares estimates of the $M_0, \ldots M_5$, described in §SupM2, are obtained and $M_0, \ldots M_3$ are converted into Preston coefficients, $c_0, \ldots c_3$, using (SEq5). Both of these sets of parameters from part of the output. Along with the actual length of the egg they provided all that is needed to derive further characteristics.

The error, as defined by (6), for the four-parameter Preston fit (i.e. the one based on $c_0, \ldots c_3$) is also part of the output.

The program `Indices.R` takes a file containing output from the first program and produces egg volume, Elongation, Pointedness, Polar Asymmetry, and R_B which is the radius of the largest circle at the blunt end (on the length scale where the egg length is two). This is sufficient information to compute the indices in Table 1. The output also indicates those eggs where the pole with the smaller circle is nearer to the latitude of maximum breadth. The egg volume is obtained from equation (SEq4), with $J = 5$. The Preston coefficients allow a smooth egg outline to be computed, and from this the point where the meridian is a maximum can be found. The egg has its widest breadth there, and so the ratio of breadth to length can be obtained and the distance of this point from one end. Then the largest circles that can fit inside the fitted egg shape and touch one of the poles are found by a search. The ratio of their diameters give Polar Asymmetry.

SupM6 Further comparison of fitting methods

Fig. SF1 compares the errors from Preston's four-parameter proposal in equation (2) with the four-parameter extensions of the proposals of Carter (1968), Baker (2002) and Troscianko (2014) introduced in the present study, in equations (SEq2), (8) and (7), respectively. All methods have comparable errors, as might be anticipated since each have the same number of parameters to adjust in arriving at a good fit. Note however that the four-parameter proposal of Carter & Morley Jones (1970), included in Fig. 5, is markedly less good, so four parameters is not necessarily enough to ensure a high quality fit.

Fig. SF2 compares each of the proposals with Preston's. For each egg and each of the alternative methods the error was divided by the Preston error. To deal with the large range of values, the logarithm of this ratio is plotted, so that a value below zero corresponds to another method giving a smaller error than Preston's. This plot confirms the interpretation of Figs 5 and SF1 as showing that Preston's method is more accurate than the other previously proposed methods.

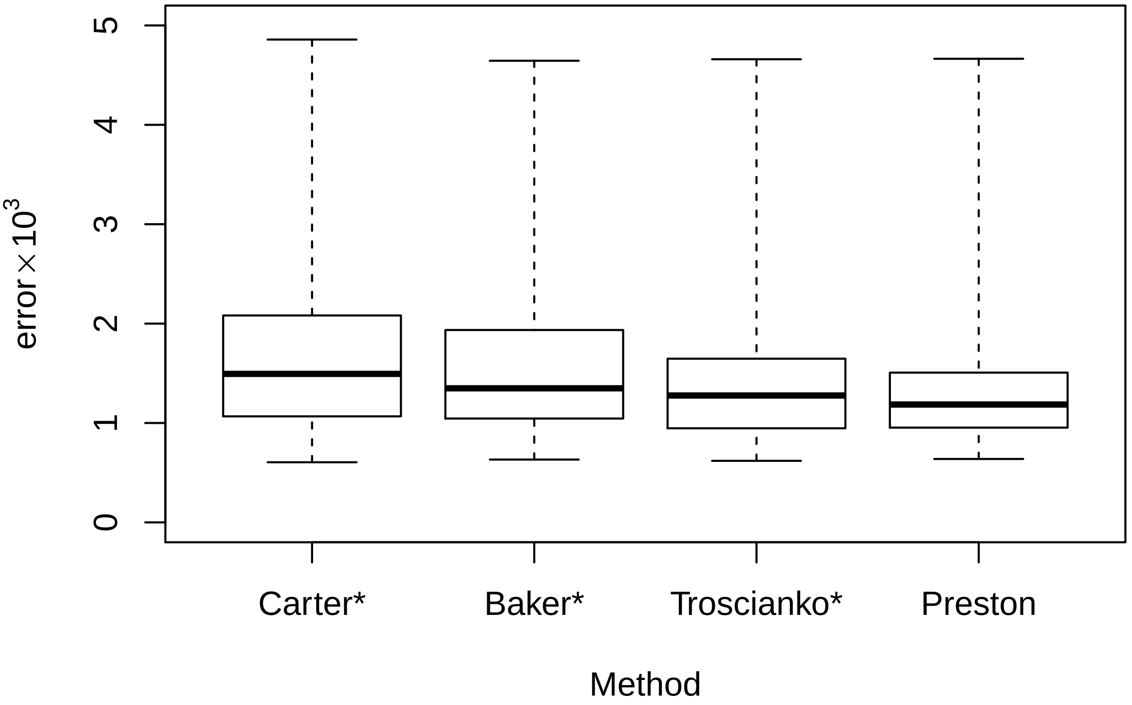

Figure SF1: Boxplots comparing the error defined at (6) (multiplied by 1000) for Preston's proposal, given in (2), and four-parameter versions of the proposals of Carter (1968), Baker (2002) and Troscianko (2014), introduced in the present study, in equations (SEq2), (8) and (7), respectively, and indicated with a '*'. The results are for the 132 eggs used also in Fig. 5. The heavy line is the median, the boxes extend between the upper and lower quartiles, the whiskers extend to the minimum and maximum.

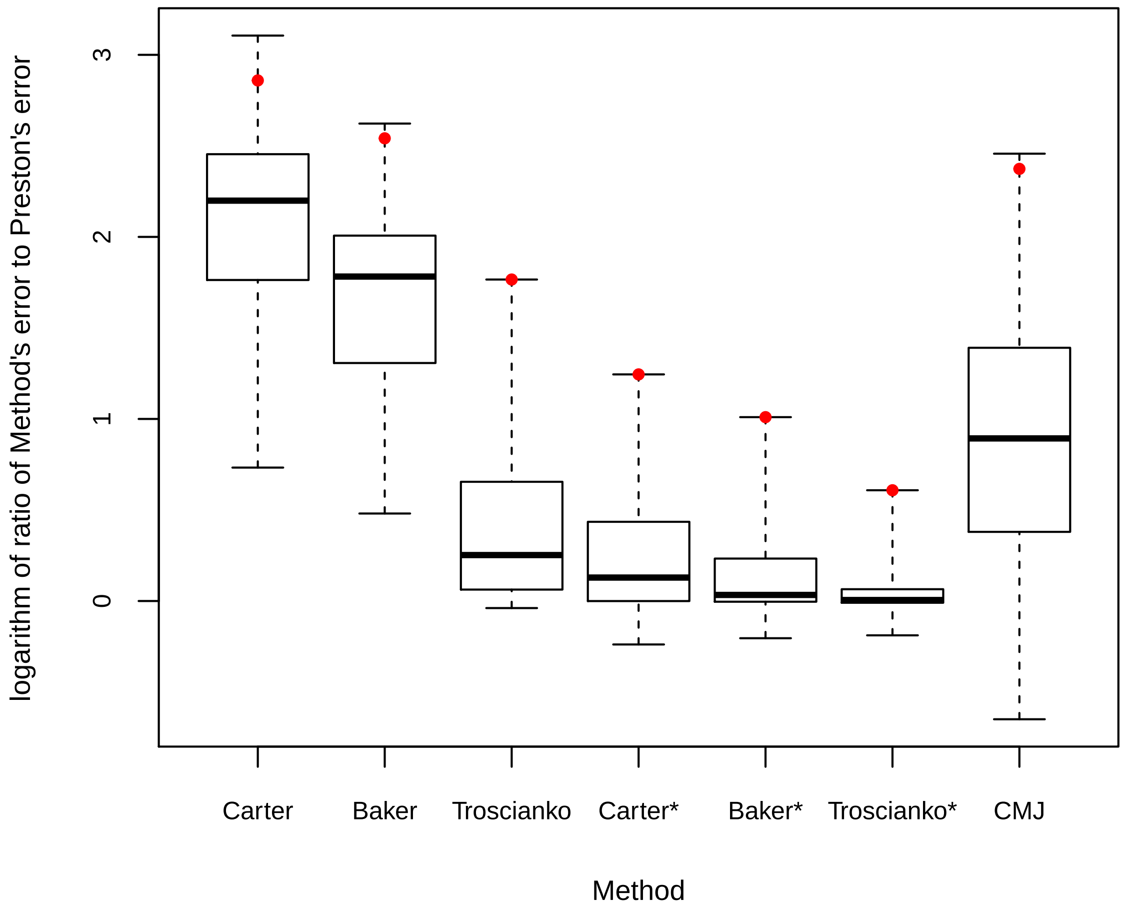

Figure SF2: Boxplots comparing the error, defined at (6), from Preston's method using equation (2), with: the proposals of Carter (1968), Baker (2002) and Troscianko (2014), given in equations (SEq1), (4) and (5); with their four-parameter variants introduced in the present study, in equations (SEq2), (8) and (7), indicated with a '*'; and with the proposal of Carter & Morley Jones (1970), decribed in equation (SEq6) and labelled CMJ. For each egg, the logarithm of the ratio of the method's error to the Preston error was obtained. The results are for the 132 eggs used also in Fig. 5. The heavy line is the median,

the minimum and maximum. The points are for the common Guillemot egg C126, used to illustrate the fit in Figs 3, 4, SF12 and SF13.

SupM7 Egg position matters

Many eggs do not rest naturally in a horizontal position. A pointed egg that is at rest will have its pointed end lower and its blunt end higher than would be the case if it were horizontal. Thus the length will be foreshortened, and so Elongation and Pointedness will have a tendency to be underestimated for more pointed eggs if a picture of a resting egg is used rather than one that is horizontal. Foreshortening should usually increase the estimate of the volume, since the larger part of the egg is more prominent. To explore these effects empirically, data on 193 eggs of various species that were photographed in both the horizontal and in their resting position were obtained. Eggs where the ratio of their actual measurements did not agree with the ratio from the horizontal photograph were excluded, leaving 185 eggs.

Fig. SF3 gives the percentage errors for four characteristics (Elongation, Pointedness, Polar Asymmetry, volume) for photographs of eggs at rest compared to the same egg when horizontal. These percentage errors are plotted against the value for the egg when horizontal. The results show that Elongation and Pointedness are generally underestimated in photographs of eggs at rest, volume overestimated and that Polar Asymmetry has considerable errors of either sign. Fig. SF4 plots the difference between the four Preston parameters when the egg is resting and when it is horizontal against the value when it is horizontal. Again clear biases are seen, with c_0 and c_1 being generally increased and c_2 and c_3 being more often decreased. Finally Fig. SF5 provides a plot of the errors in Stoddard et al.'s (2017) 'ellipticity' and 'asymmetry', which are sometimes considerable.

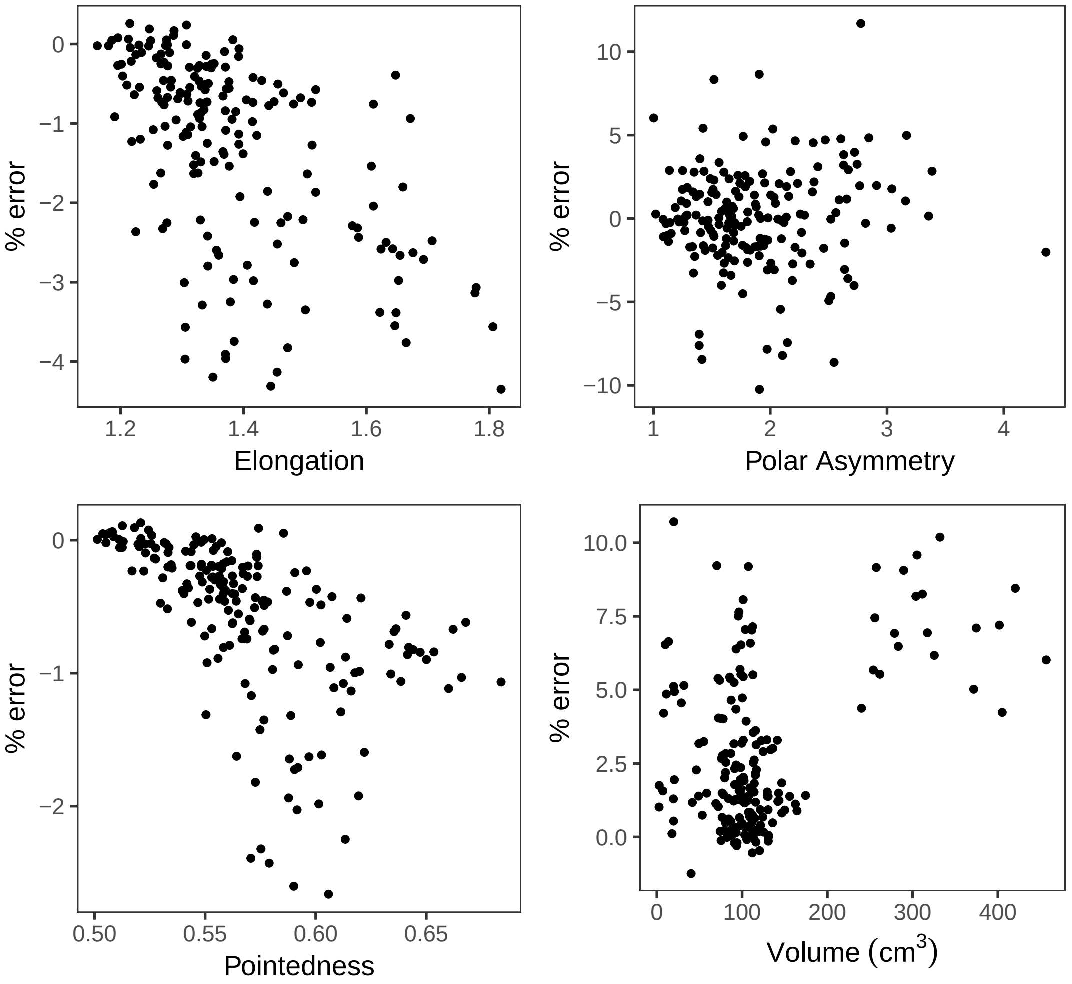

Figure SF3: For each of the four characteristics (Elongation, Pointedness, Polar Asymmetry, volume), the percentage error from a photograph in a resting position is plotted against the value of the characteristic for a photograph in a horizontal position.

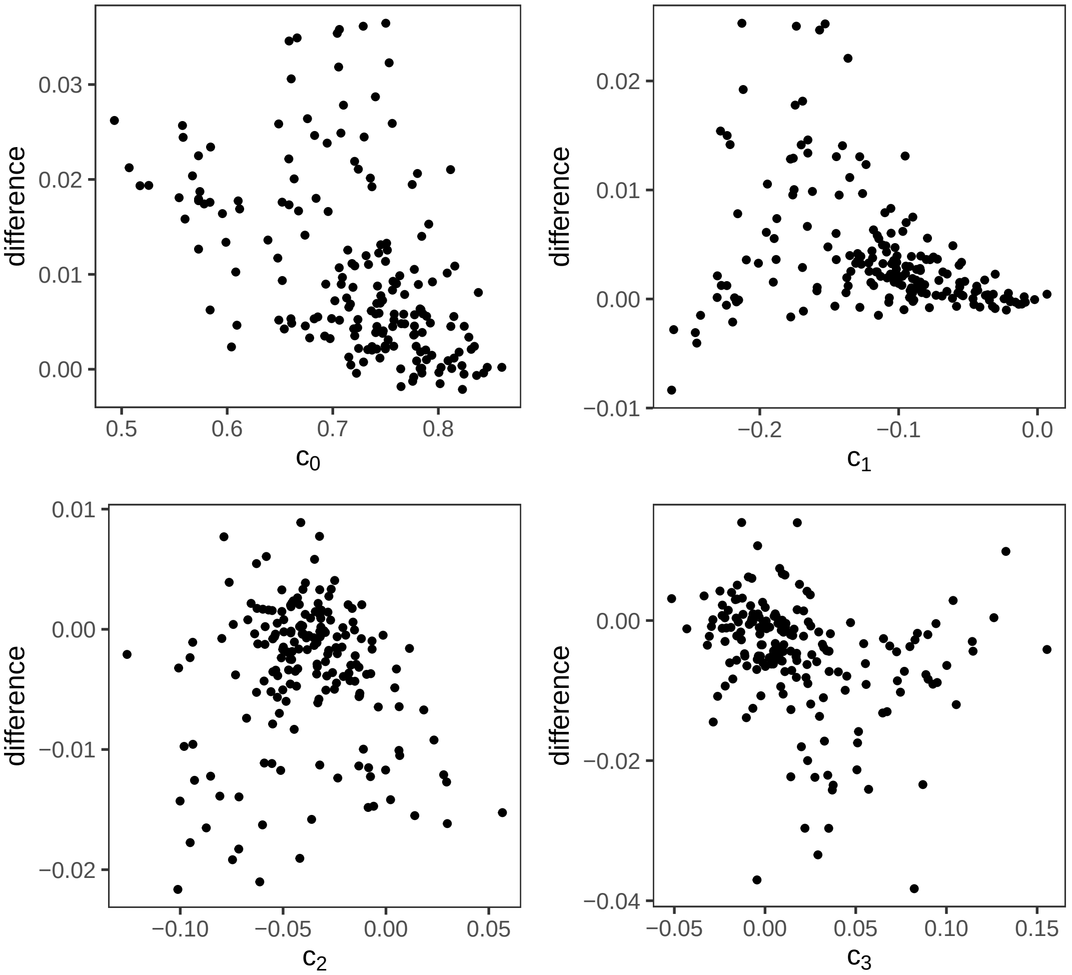

Figure SF4: For each of the four Preston parameters, the difference in the value between the egg photographed in a resting position and in a horizontal position is plotted against the value of the parameter from the photograph in a horizontal position

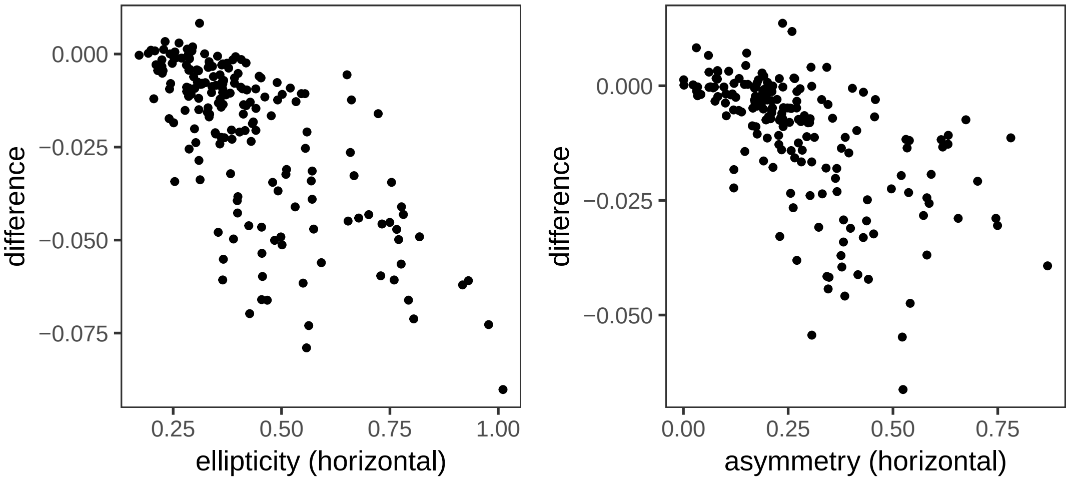

Figure SF5: For Stoddard et al.'s (2017) ellipticity and asymmetry, the difference in the value between the egg photographed in a resting position and in a horizontal position is plotted against the value from the photograph in a horizontal position.

SupM8 Relations between indices

The relation between indices will depend on the collection of eggs used. Here there is a particular focus on pyriform eggs and so the relationship between the indices will be examined using collections of eggs from three species (735 *Uria aalge*, 296 *Uria lomvia* and 101 *Alca torda*) for which Preston parameters have been obtained. The two *Uria* species produce pyriform eggs; the eggs of the closely related razorbill are not pyriform and have been described† as 'elliptical ovate' to 'elongate ovate' in shape.

As is indicated in §2.7, it is straightforward to compute various indices from Table 1 for these eggs, since all the measurements indicated in Fig. 2 can be derived from the Preston four-parameter fit, as can Stoddard et al.'s (2017) indices. As noted previously, Preston's c_0 is the equatorial diameter and so Stoddard et al.'s (2017) 'ellipticity' should be very similar to $(1/c_0) - 1$. To confirm this, the index Preston.ellipticity is defined to be $(1/c_0) - 1$ and included in the comparisons.

For indices, the correct indication of their relationships is through their Spearman correlations (since an index is not changed in an important way by monotonic transformations). Fig. SF6 gives both a graphical illustration and numerical values for the correlation matrix for the 735 *Uria aalge* eggs. The indices are in the order given in Table 1, with Preston.ellipticity inserted at a natural place. A feature of this matrix is that four of the indices — Mytiai & Matsyura's (2017) infundibular, cloacal, complementarity and Mänd et al.'s (1986) sharp-end convexity — have generally negative correlations with other indices. Thus, in rough terms, they get smaller when the other indications of departures from a circle are getting larger. To remedy this, a slight modification of each of these indices is proposed. For sharp-end convexity the new index is -1 times the original index. For infundibular, cloacal and complementarity the new index is the reciprocal of the original one. These modifications change the signs of Spearman correlations but not their absolute values. Fig. SF7 gives the correlations for this new collection, where a '1' is added

†Bent, A. C. (1919), *Life Histories of North American Diving Birds*, General Publishing Company, Toronto

to the name of those indices that are redefined. The order of the indices has also been changed to group together those with higher (positive) correlations.

The shape of the correlation matrix in Fig. SF7 shows four groups of indices, indicated by the high correlations near the diagonal. We may identify indices that represents these groups. The first corresponds to Elongation, the second to Preston's (1968) bicone, the third to Pointedness and the fourth to Polar Asymmetry. Thus, for the complexities of pyriform shape, just as four parameters are needed for the Preston fit, four shape indices capture different aspects of their shape. Of these four, Preston's (1968) bicone is rather different from the other three, in that it is an index of the average curvature at the two poles, and seems less directly related to the main features of the shape. The other three provide a satisfactory basis for comparisons of pointedness in a general sense.

The correlation matrices for the 296 *Uria lomvia* and 101 *Alca torda* are displayed in Figs SF8 and SF9. They show broadly the same features as Fig. SF7, except that for the *Alca torda* eggs, which are not markedly pyriform, the separation of the third and fourth group of indices (corresponding to Pointedness and Polar Asymmetry) is less pronounced. This is expected, as a simpler description of shape should suffice for eggs that are not pyriform.

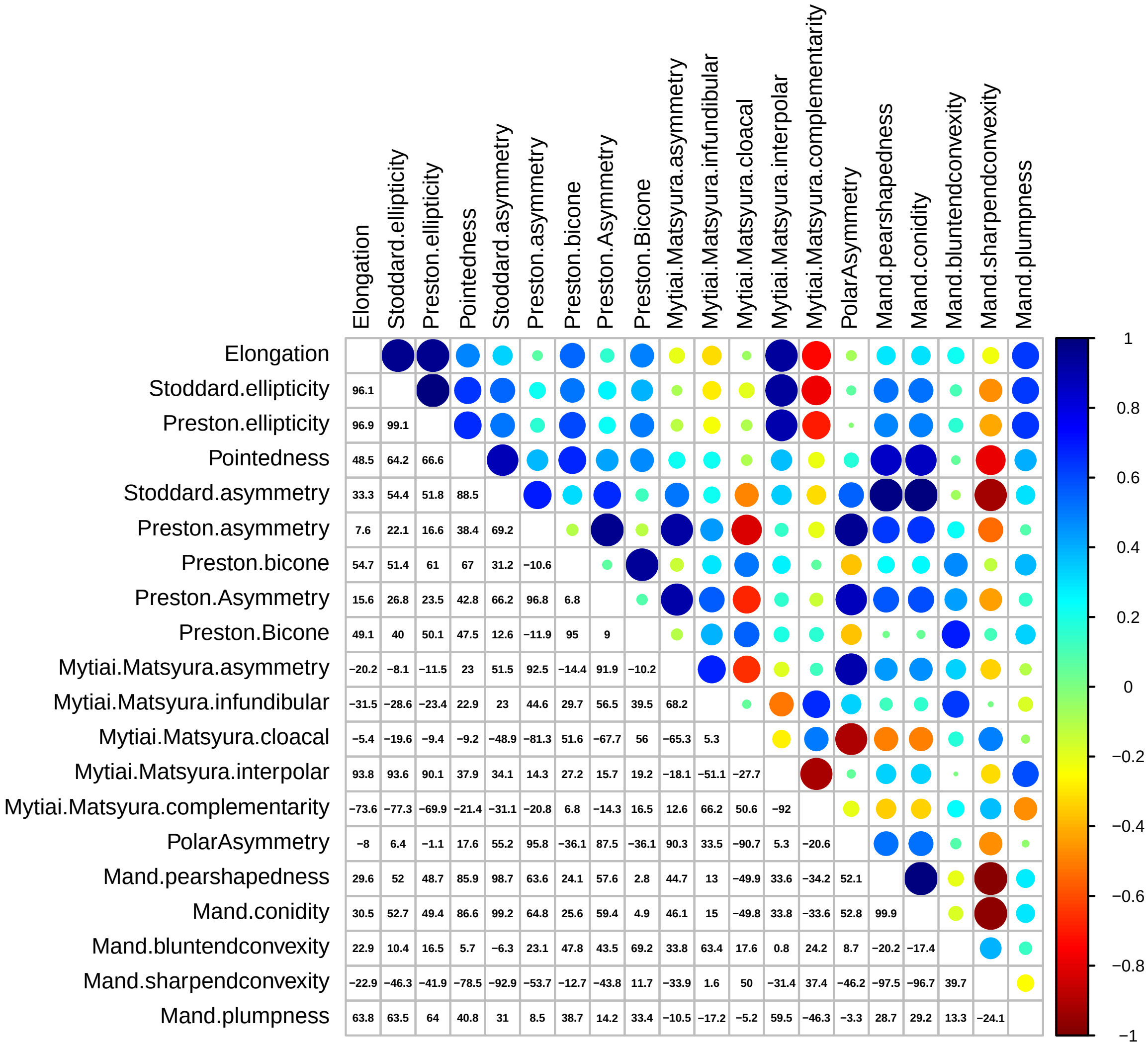

Figure SF6: For the photographs of 735 *Uria aalge* eggs, Spearman correlation between various indices described in Table 1 and also Preston.ellipticity, given by $(1/c_0) - 1$ where c_0 is the first Preston parameter. Values are represented by colour and circle size in the upper triangle; the numerical values (times 100) are given in the lower triangle.

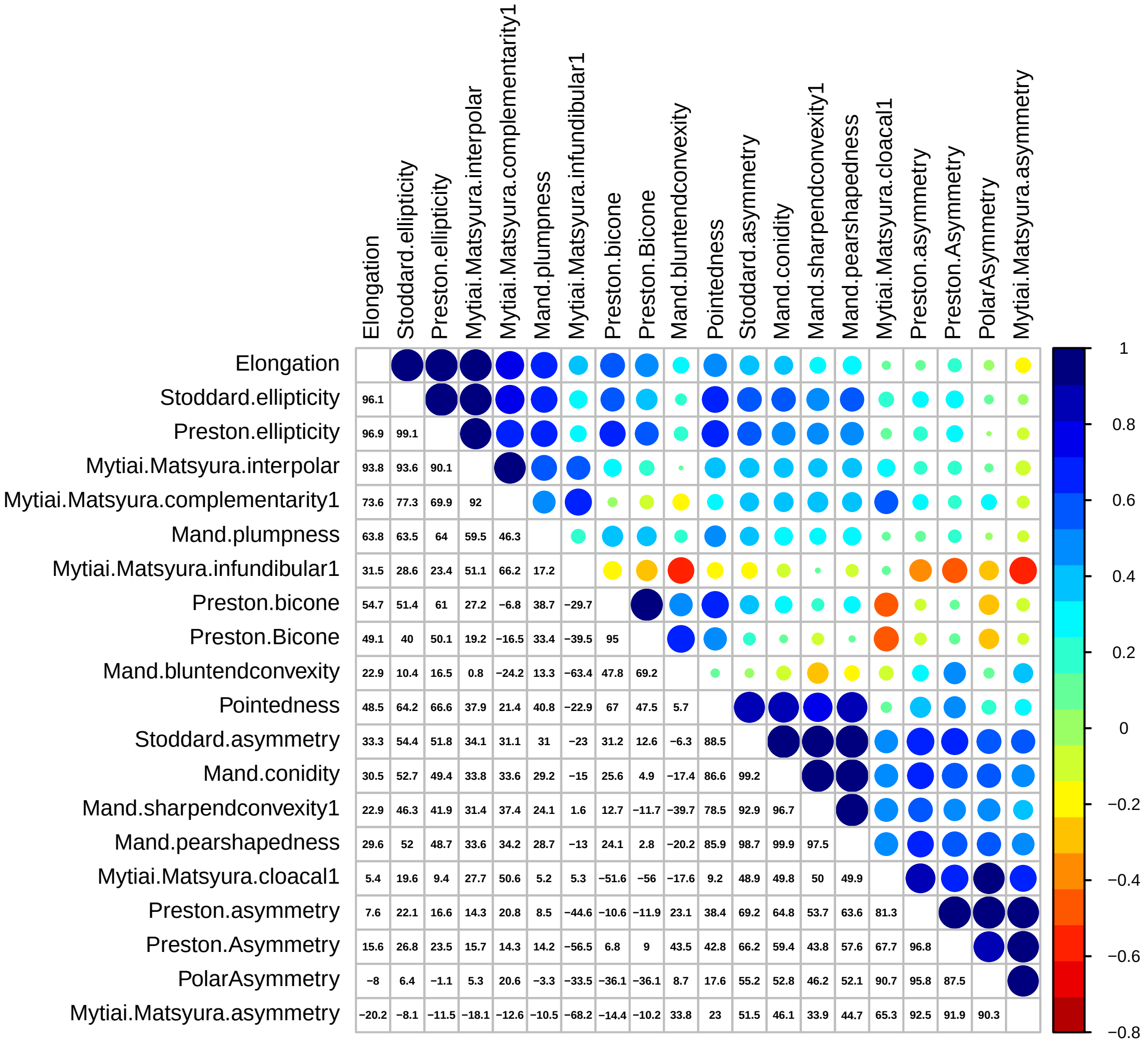

Figure SF7: For the photographs of 735 *Uria aalge* eggs, Spearman correlation between various indices. The indices are as in Fig. SF6 with the following exceptions. Mand.sharpendconvexity1 is −1 times the original index, and Mytiai.Matsyura.infundibular1, Mytiai.Matsyura.cloacal1 and Mytiai.Matsyura.complementarity1 are the reciprocals of the original index. Values are represented by colour and circle size in the upper triangle; the numerical values (times 100) are given in the lower triangle.

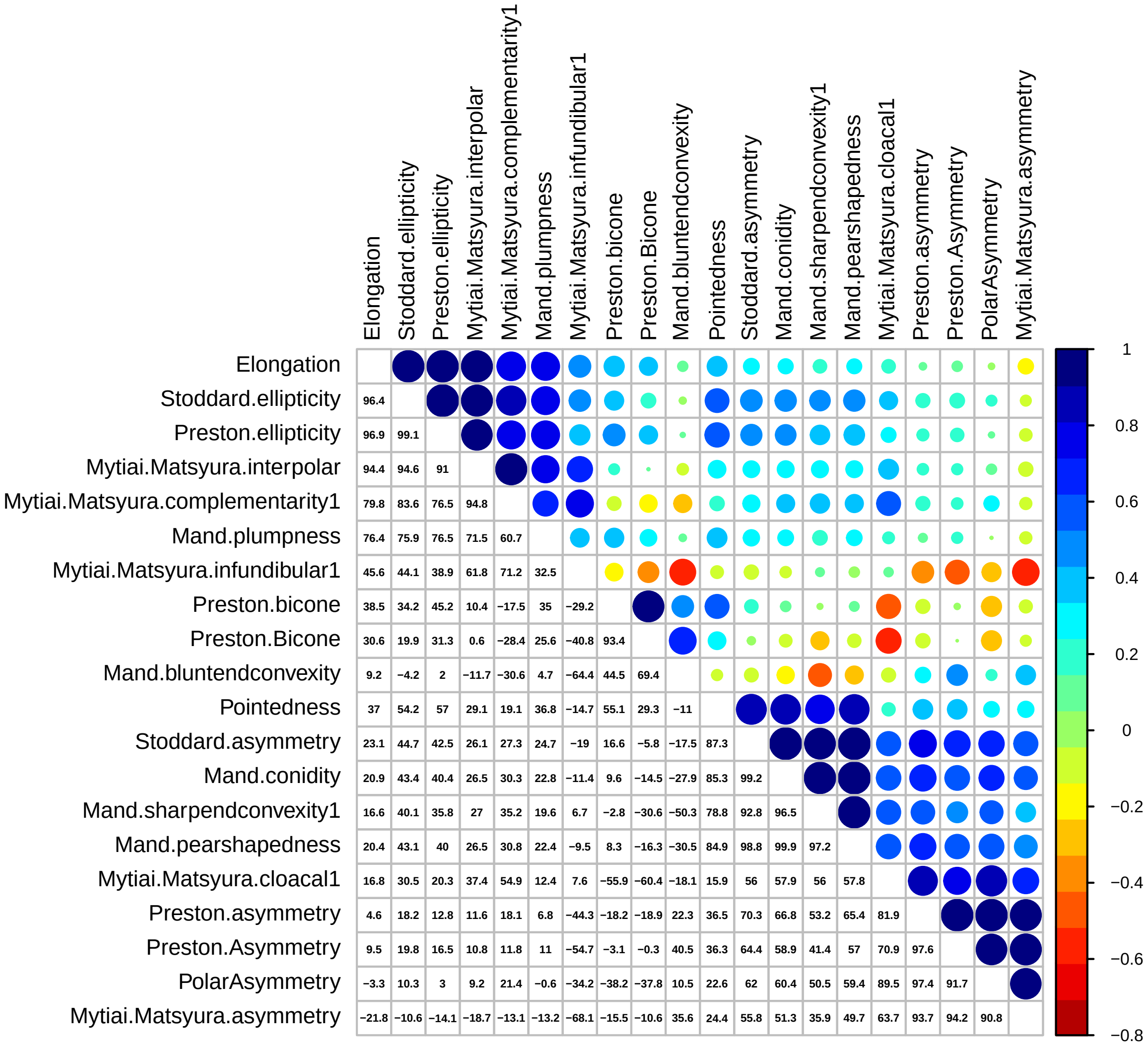

Figure SF8: For the photographs of 296 *Uria lomvia* eggs, Spearman correlation between various indices. The indices are as in Fig. SF6 with the following exceptions. Mand.sharpendconvexity1 is -1 times the original index, and Mytiai.Matsyura.infundibular1, Mytiai.Matsyura.cloacal1 and Mytiai.Matsyura.complementarity1 are the reciprocals of the original index. Values are represented by colour and circle size in the upper triangle; the numerical values (times 100) are given in the lower triangle.

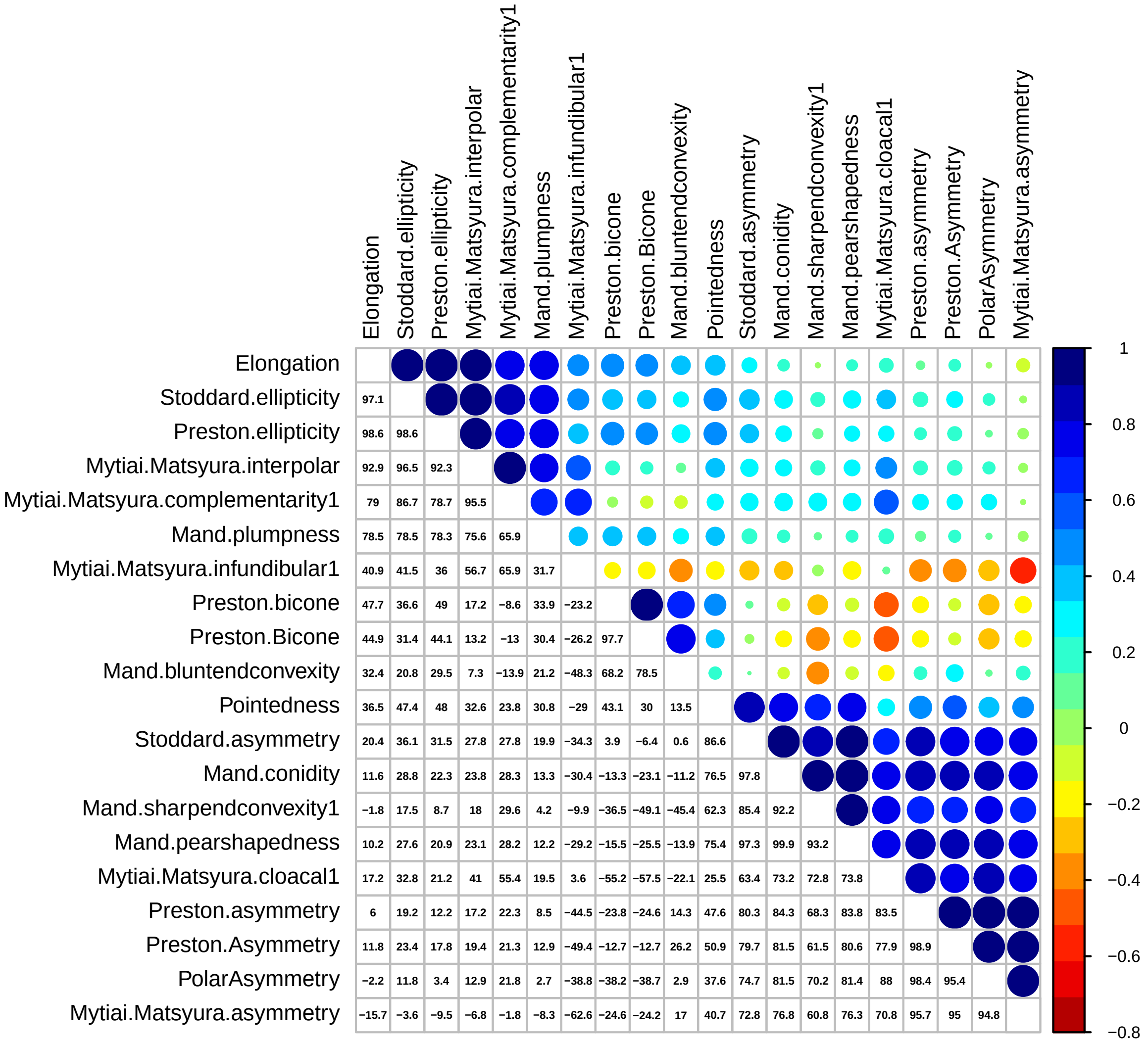

Figure SF9: For the photographs of 101 *Alca torda* eggs, Spearman correlation between various indices. The indices are as in Fig. SF6 with the following exceptions. Mand.sharpendconvexity1 is −1 times the original index, and Mytiai.Matsyura.infundibular1, Mytiai.Matsyura.cloacal1 and Mytiai.Matsyura.complementarity1 are the reciprocals of the original index. Values are represented by colour and circle size in the upper triangle; the numerical values (times 100) are given in the lower triangle.

SupM9 Additional illustrations for Egg C126

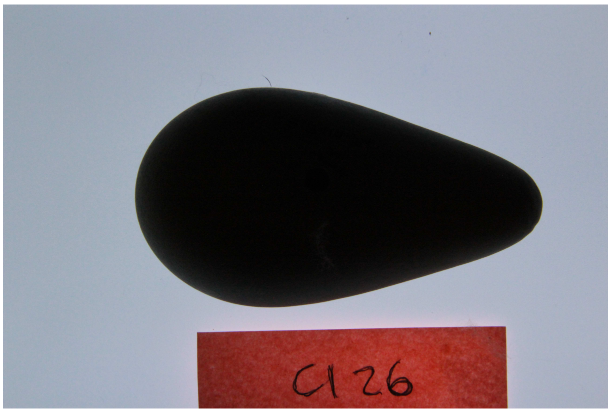

Figure SF10: The photograph of guillemot egg C126

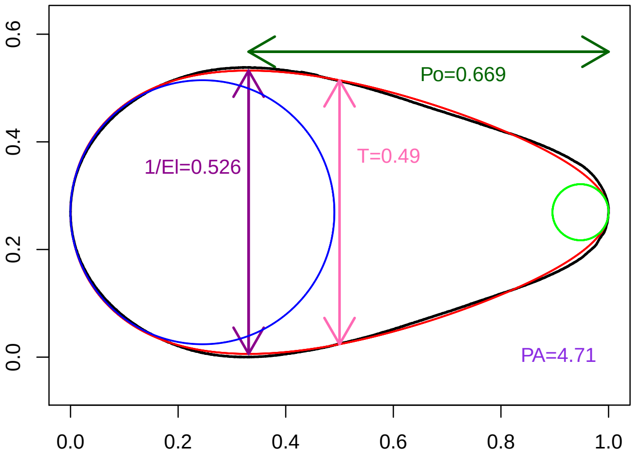

Figure SF11: The actual egg shape of C126 is the black outline. The Troscianko fit is in red. The error, as defined at equation (6), is 0.00534. The values of Po, 1/El and T based on the Troscianko fit are indicated. The Troscianko PA value(4.71) is markedly larger than the value of 2.63 obtained from the Preston fit (see Fig. 3) because the Troscianko fit is too pointed at the pointed end.

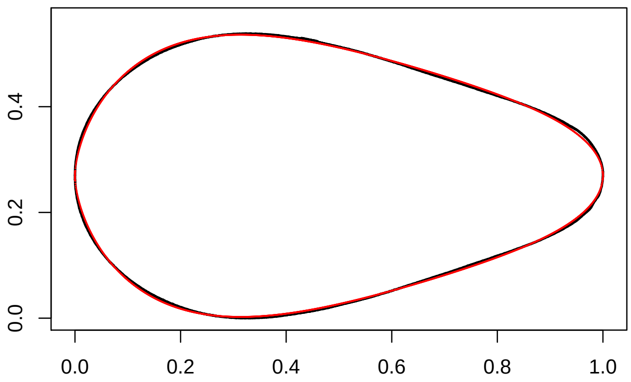

Figure SF12: The actual egg shape is the black outline; the 4 parameter Carter-fit, based on equation (SEq2), is in red.

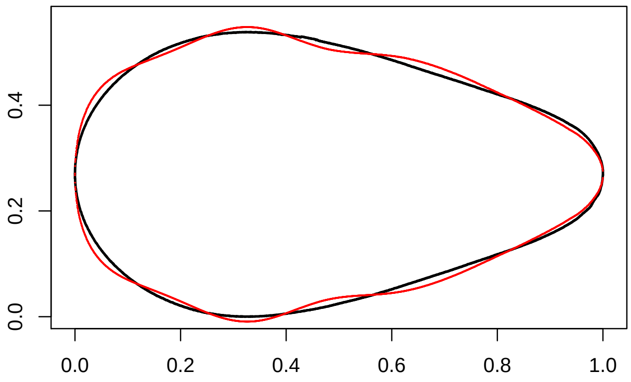

Figure SF13: Fit using the method from Carter & Morley Jones (1970) in red, illustrating the undulating fitted meridian.
